# Loss of DRC1 function leads to multiple morphological abnormalities of the sperm flagella and male infertility in human and mouse

**DOI:** 10.1093/hmg/ddab171

**Published:** 2021-06-24

**Authors:** Jintao Zhang, Xiaojin He, Huan Wu, Xin Zhang, Shenmin Yang, Chunyu Liu, Siyu Liu, Rong Hua, Shushu Zhou, Shuqin Zhao, Fan Hu, Junqiang Zhang, Wangjie Liu, Huiru Cheng, Yang Gao, Feng Zhang, Yunxia Cao, Mingxi Liu

**Affiliations:** State Key Laboratory of Reproductive Medicine, Department of Histology and Embryology, School of Basic Medical Sciences, Nanjing Medical University, Nanjing 211166, China; Reproductive Medicine Center, Department of Obstetrics and Gynecology, the First Affiliated Hospital of Anhui Medical University, Hefei 230022, China; NHC Key Laboratory of Study on Abnormal Gametes and Reproductive Tract, Anhui Medical University, Hefei 230032, China; Reproductive Medicine Center, Department of Obstetrics and Gynecology, the First Affiliated Hospital of Anhui Medical University, Hefei 230022, China; NHC Key Laboratory of Study on Abnormal Gametes and Reproductive Tract, Anhui Medical University, Hefei 230032, China; State Key Laboratory of Reproductive Medicine, Department of Histology and Embryology, School of Basic Medical Sciences, Nanjing Medical University, Nanjing 211166, China; State Key Laboratory of Reproductive Medicine, Center for Reproduction and Genetics, Suzhou Hospital Affiliated to Nanjing Medical University, Suzhou 215002, China; Obstetrics and Gynecology Hospital, NHC Key Laboratory of Reproduction Regulation (Shanghai Institute for Biomedical and Pharmaceutical Technologies), State Key Laboratory of Genetic Engineering at School of Life Sciences, Fudan University, Shanghai 200011, China; Shanghai Key Laboratory of Female Reproductive Endocrine Related Diseases, Shanghai 200011, China; State Key Laboratory of Reproductive Medicine, Department of Histology and Embryology, School of Basic Medical Sciences, Nanjing Medical University, Nanjing 211166, China; State Key Laboratory of Reproductive Medicine, Department of Histology and Embryology, School of Basic Medical Sciences, Nanjing Medical University, Nanjing 211166, China; State Key Laboratory of Reproductive Medicine, Department of Histology and Embryology, School of Basic Medical Sciences, Nanjing Medical University, Nanjing 211166, China; State Key Laboratory of Reproductive Medicine, Animal Core Facility of Nanjing Medical University, Nanjing 211166, China; State Key Laboratory of Reproductive Medicine, Nanjing Medical University, Nanjing 211166, China; NHC Key Laboratory of Study on Abnormal Gametes and Reproductive Tract, Anhui Medical University, Hefei 230032, China; Key Laboratory of Population Health Across Life Cycle, Anhui Medical University, Ministry of Education of the People’s Republic of China, Hefei 230032, China; Obstetrics and Gynecology Hospital, NHC Key Laboratory of Reproduction Regulation (Shanghai Institute for Biomedical and Pharmaceutical Technologies), State Key Laboratory of Genetic Engineering at School of Life Sciences, Fudan University, Shanghai 200011, China; Shanghai Key Laboratory of Female Reproductive Endocrine Related Diseases, Shanghai 200011, China; NHC Key Laboratory of Study on Abnormal Gametes and Reproductive Tract, Anhui Medical University, Hefei 230032, China; Key Laboratory of Population Health Across Life Cycle, Anhui Medical University, Ministry of Education of the People’s Republic of China, Hefei 230032, China; Reproductive Medicine Center, Department of Obstetrics and Gynecology, the First Affiliated Hospital of Anhui Medical University, Hefei 230022, China; NHC Key Laboratory of Study on Abnormal Gametes and Reproductive Tract, Anhui Medical University, Hefei 230032, China; Obstetrics and Gynecology Hospital, NHC Key Laboratory of Reproduction Regulation (Shanghai Institute for Biomedical and Pharmaceutical Technologies), State Key Laboratory of Genetic Engineering at School of Life Sciences, Fudan University, Shanghai 200011, China; Shanghai Key Laboratory of Female Reproductive Endocrine Related Diseases, Shanghai 200011, China; Reproductive Medicine Center, Department of Obstetrics and Gynecology, the First Affiliated Hospital of Anhui Medical University, Hefei 230022, China; NHC Key Laboratory of Study on Abnormal Gametes and Reproductive Tract, Anhui Medical University, Hefei 230032, China; Key Laboratory of Population Health Across Life Cycle, Anhui Medical University, Ministry of Education of the People’s Republic of China, Hefei 230032, China; State Key Laboratory of Reproductive Medicine, Department of Histology and Embryology, School of Basic Medical Sciences, Nanjing Medical University, Nanjing 211166, China

## Abstract

Motile cilia and flagellar defects can result in primary ciliary dyskinesia, which is a multisystemic genetic disorder that affects roughly 1:10 000 individuals. The nexin-dynein regulatory complex (N-DRC) links neighboring doublet microtubules within flagella, serving as a central regulatory hub for motility in Chlamydomonas. Herein, we identified two homozygous *DRC1* variants in human patients that were associated with multiple morphological abnormalities of the sperm flagella (MMAF) and male infertility. *Drc1*^−/−^, *Drc1*^R554X/R554X^ and *Drc1*^W244X/W244X^ mice on the C57BL/6 background suffered from pre-pubertal mortality. However, when the ICR background was introduced, some of these mice were able to survive and recapitulate the MMAF phenotypes detected in human patients. By analyzing these animals, we determined that DRC1 is an essential regulator of N-DRC assembly in cilia and flagella. When DRC1 is absent, this results in the shortening of cilia and consequent impairment of their motility. Damage associated with DRC1 deficiency in sperm flagella was more pronounced than in cilia, as manifested by complete axoneme structural disorder in addition to the loss of the DRC structure. Altogether, these findings suggest that DRC1 is required for the structural stability of flagella but not cilia, emphasizing the key role of this protein in mammalian species.

## Introduction

Flagella and motile cilia are evolutionarily ancient structures present in prokaryotic and eukaryotic cells that are involved in sensation and movement (1). These organelles also play essential roles in the development and functionality of key systems including the respiratory, nervous and reproductive systems (2). Genetic defects in the motility of flagella and cilia can cause a multisystem disorder known as primary ciliary dyskinesia (PCD) (3,4), which affects roughly 1:10 000 individuals globally (5,6). The nexin-dynein regulatory complex (N-DRC) functions by linking neighboring doublet microtubules within motile cilia and flagella, stabilizing the axonemal core structure and thereby regulating ciliary motility (7–13). In the flagella of Chlamydomonas cells, at least 11 subunit proteins compose the N-DRC (7,14), and many homologs of these proteins are present in humans and mice. The deletion of DRC genes in mice can result in an array of distinct phenotypes. For example, *Drc7* (15) and *Drc9* (16) knockout mice exhibit multiple morphological abnormalities of the sperm flagella (MMAF) and male infertility, whereas *Drc5* deletion (17) causes asthenospermia and the deletion of *Drc6* (15) does not induce significant flagellar abnormalities. A single mutation at amino acid position 89 (Leu89Pro) in *DRC3* (*LRRC48*) can result in classical symptoms of PCD such as mucus accumulation, male sterility and an increased risk of postnatal death (18).

Analyses of Chlamydomonas have identified DRC1, DRC2 and DRC4 as core N-DRC structural components (11). In mice, the loss of DRC4 expression is associated with severe hydrocephaly and lethality within 14–21 days after birth (19). This suggests that this core N-DRC protein is essential for murine survival. No mice harboring knockout mutations for core DRC genes that survive into adulthood have been described to date. However, one case report published in 2013 described the case of a PCD patient exhibiting a homozygous nonsense mutation (c.2056A > T) in *DRC1* that was predicted to cause the pre-mature translational arrest of the nascent DRC1 peptide (p.Lys686*) (20). That patient suffered from symptoms including chronic otitis media, sinusitis, recurrent pneumonia and neonatal respiratory distress, all of which are consistent with the diagnosis of PCD. In another recent study, a patient with diffuse panbronchiolitis was found to harbor a large homozygous deletion spanning exons 1–4 of *DRC1* (21). In both of these cases, the patients survived to adulthood. No *Drc1* knockout mice have yet been developed to explore the functional role of this protein in mammals to date.

Herein, we found that two homozygous *DRC1* mutations resulted in MMAF and male infertility phenotypes in humans without causing any concomitant respiratory symptoms. While *Drc1*^−/−^, *Drc1*^R554X/R554X^ and *Drc1*^W244X/W244X^ mice on the C57BL/6 background suffered from pre-pubertal death in all cases, the introduction of the ICR background into these strains enabled some of the mice to survive and to exhibit development and behavior similar to that of wild-type (WT) control mice. The surviving mice recapitulated the MMAF phenotypes observed in the context of human DRC1 deficiency. Interestingly, we further found that damage to sperm flagellum was more severe than ciliary damage in *Drc1*^R554X/R554X^ and *Drc1*^W244X/W244X^ mice, resulting in damage beyond just the loss of the DRC structure. Altogether, our data suggest that *DRC1* mutations can result in dysregulated cilia and flagella formation in humans and mice while also indicating that this protein regulates the structural homeostasis of sperm flagella.

## Results

### Exome sequencing reveals the presence of homozygous *DRC1* variants in MMAF patients

**Figure 1:**
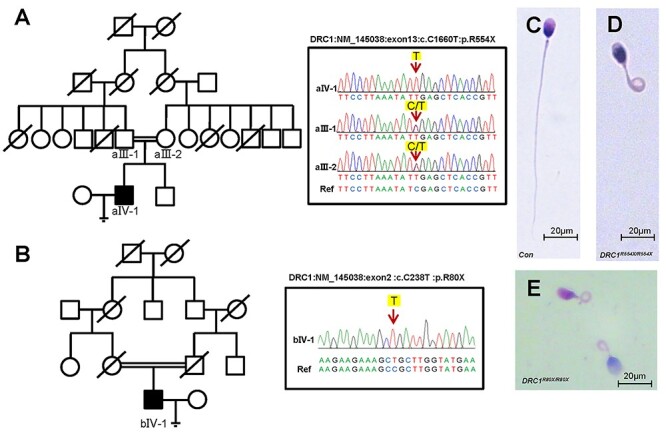
Biallelic mutations in *DRC1* were identified in probands with MMAF. (**A** and **B**) Biallelic mutations in DRC1 were identified in the probands from two families. The mutations identified by WES were further verified by Sanger sequencing. Red arrows indicate the positions of point mutations. (**C**) Light microscopy revealed a spermatozoon with normal morphology from a healthy male. (**D** and **E**) Most spermatozoa of DRC1-mutated probands (D: *DRC1*^R554X/R554X^; **F**: *DRC1*^R80X/R80X^) exhibited flagellar morphological abnormalities.

Exome sequencing represents a powerful approach to exploring the genetic basis for flagellar defects in sperm, and at least 19 genes associated with MMAF phenotypes have been identified to date (22–40). Of our overall 100 MMAF patient cohort, we identified 76 patients with harmful variants in known MMAF-related genes. After re-analyzing exome data from the remaining patients, we detected two unrelated patients harboring homozygous variants in *DRC1*. Patient aIV-1 harbored a stop codon gain variant c.C1660T: p.R554X (NM_145038.5) (Fig. 1A; *DRC1*^R554X/R554X^) not recorded in the Genome Aggregation Database, while patient bIV-1 harbored a distinct stop-gain variant c.C238T: p.R80X (NM_145038.5) (Fig. 1B; *DRC1*^R80X/R80X^) with a minor allele frequency of 3.19e^−5^ in the Genome Aggregation Database. Morphological analyses confirmed that sperm from both patients exhibited characteristic MMAF features (Fig. 1C and D, Table 1).

**Table 1:** Clinical characteristics of the subjects carrying homozygous stop-gain mutation in *DRC1*

Clinical characteristics	aIV-1	bIV-1
Semen parameters		
Ejaculated sperm volume (ml)	2.3	2.8
Seminal PH	7.3	7.4
Ejaculated sperm concentration (million/ml)	11.3	8.84
Progressive motility	0.5%	0%
Sperm morphology		
Abnormal head	87%	100%
Abnormal flagella	99%	99%
Serum sex hormone levels		
FSH (mIU/ml)	4.01	ND
LH (mIU/ml)	4.39	ND
T (nmol/l)	8.41	ND
PRL (ng/ml)	7.53	ND
Karyotype	46XY 46XX	ND
AZFs deletion	Undetectable	Undetectable

**Figure 2:**
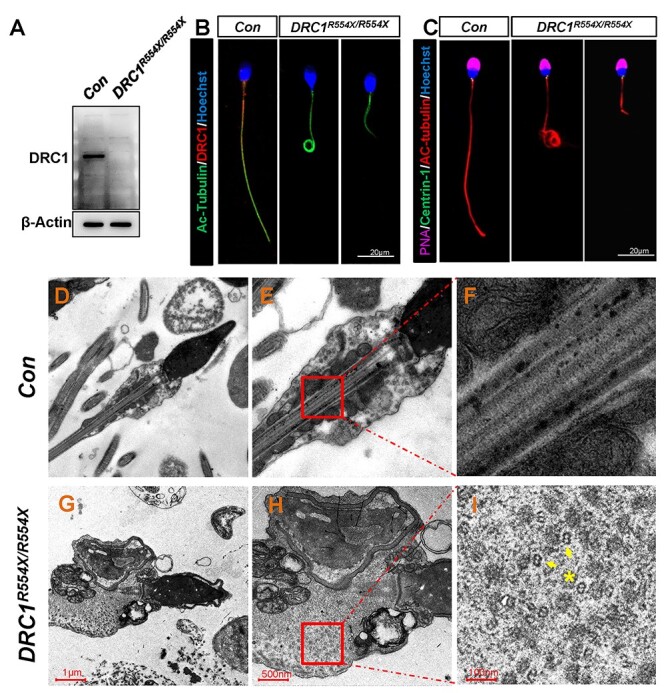
The *DRC1*^R554X/R554X^ mutation causes multiple morphological abnormalities and ultrastructural disorder in sperm flagella. (**A**) DRC1 is missing in spermatozoa from a *DRC1*^R554X/R554X^ mutant individual. (**B**) Spermatozoa from a fertile control individual and from a *DRC1*^R554X/R554X^ mutant individual were stained with anti-DRC1 and anti-Ac-Tubulin. (**C**) Spermatozoa from a fertile control individual and from a *DRC1*^R554X/R554X^ mutant individual were stained with anti-Centrin-1, anti-Ac-Tubulin and PNA. (**D**–**F**) The normal axoneme is composed of nine doublets of microtubules circularly arranged around a central-pair complex of microtubules (9 + 2 organization). (**G**–**I**) The *DRC1*^R554X/R554X^ mutation is associated with severe axonemal disorganization and evidence of unassembled microtubule doublets.

We next generated antisera specific for the homologous N-terminal (1–146 aa) region of human and murine DRC1, which we used to determine that no DRC1 protein was detectable in the sperm of patient aIV-1 (Fig. 2A), consistent with nonsense-mediated mRNA decay (NMD) (41–43). Immunofluorescence analyses similarly confirmed the absence of DRC1 in sperm from this patient, while Ac-tubulin staining revealed that *DRC1*^R554X/R554X^ sperm presented with short, coiled, absent or irregular flagella (Fig. 2B and C, Supplementary Material, Fig. S1A and B). The sperm of patient bIV-1 exhibited a phenotype similar to that of Patient aIV-1 (Supplementary Material, Fig. S1C). TEM analyses revealed the disordered structure of the flagellar axoneme in *DRC1*^R554X/R554X^ sperm, with microtubules being scattered in the cytoplasm and with normal centriole implantation and implantation nest formation (Fig. 2D–I). Axoneme structural disorder was also evident in *DRC1*^R80X/R80X^ sperm (Supplementary Material, Fig. S1C). Following ICSI treatment, Patient aIV-1 was able to obtain fertilized embryos, and one of which was successfully implanted (Table 2), while Patient bIV-1 did not undergo further treatment. The parents of both of these patients were closely related to one another, and the genetic characteristics of the observed *DRC1* mutations were consistent with a recessive genetic model, suggesting that a loss of human DRC1 functionality results in MMAF and male infertility.

**Table 2:** Clinical outcomes of the *DRC1*-mutated patients following ICSI

Clinical characteristics	
Male age (years)	35
Female age (years)	28
No. of ICSI cycles	1
No. of oocytes injected	7
Fertilization rate	4/7 (57.14%)
Cleavage rate	2/4 (50.0%)
Eight cells embryo development rate	2/4 (50.0%)
Blastocyst development rate	2/4 (50.0%)
No. of frozen–thawed embryos transfer cycles	1
Number of embryos transferred	2
Implantation rate (%)	1/2 (50%)
Clinical pregnancy	Y
Miscarriage	N

### *Drc1* knockout mice on a C57BL/6 background suffer from hydrocephaly and postnatal death

We next evaluated DRC1 sequence conservation among species, revealing DRC1 sequences to be relatively conserved in *Chlamydomonas reinhardtii*, *Drosophila melanogaster*, *Gallus gallus*, *Homo sapiens*, *Macaca fascicularis*, *Mus musculus*, *Rattus norvegicus* and *Xenopus laevis*. DRC1 R80 and R554 were both conserved in mice (Supplementary Material, Fig. S2). To understand the functional role of DRC1 *in vivo*, we then employed a CRISPR/Cas9 approach to generate two *Drc1* mutant mouse strains (Supplementary Material, Fig. S3). A stable *Drc1* mutant mouse line carrying a 1 bp deletion within exon 13 of this gene (Supplementary Material, Fig. S3A) was established (*Drc1^−/−^*). In addition, a mouse model harboring the R554 mutation observed in MMAF patients (*Drc1*^R554X/R554X^, Supplementary Material, Fig. S3B) was generated. Unexpectedly, mice harboring both of these mutations experienced pre-puberal death, usually before postnatal day 12 (Table 3). These mice exhibited clear signs of growth retardation and hydrocephaly (Supplementary Material, Fig. S4).

**Table 3:** Survival rate after puberty of Drc1 mutant mice in different genetic background

Genotype	Background	No. of mice with indicated genotype (Hydrocephalus)			Total	Alive after puberty
		+/+	+/−	−/−		
*Drc1* ^+/−^ *X Drc1* ^+/−^	B6	45	85	25(25)	155	0(0)
*Drc1* ^+/−^ *X Drc1* ^+/−^	B6(ICR)	43	81	32(27)	156	5(15.6%)
*Drc1* ^+/W244X^ *X Drc1* ^+/W244X^	B6	22	29	12(12)	63	0(0)
*Drc1* ^+/W244X^ *X Drc1* ^+/W244X^	B6(ICR)	76	215	62(22)	353	40(64.5%)
*Drc1* ^+/R554X^ *X Drc1* ^+/R554X^	B6	37	54	26(26)	117	0(0)
*Drc1* ^+/R554X^ *X Drc1* ^+/R554X^	B6(ICR)	92	202	58(35)	352	23(39.7%)

As the Cas9 system has the potential to introduce off-target genetic changes and these two murine lines were constructed using the same sgRNA, we additionally attempted to generate an additional murine line simulating human DRC1 mutations using base editing technology. After selecting mutation sites in the gnomAD database capable of causing a loss of DRC1 function and analyzing potential sgRNA designs and conserved residues, we determined that the W244X model would be well-suited to this base editing approach (Supplementary Material, Fig. S3). However, as with these first two mouse models, all *Drc1*^W244X/W244X^ mice died before puberty (Table 3).

### *Drc1* is necessary for male fertility and sperm flagellum formation in C57BL/6 × ICR mice

**Figure 3:**
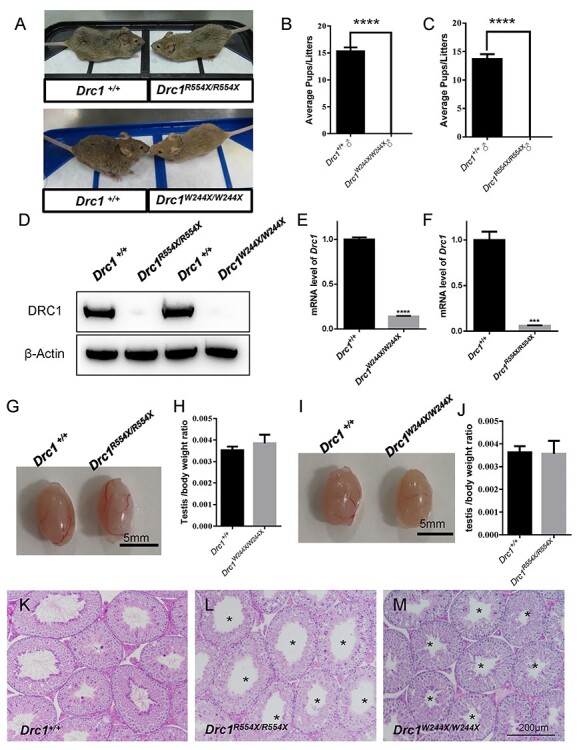
The DRC1 mutation results in male infertility in mice of the C57BL/6 × ICR background. (**A**) *Drc1*^R554X/R554X^ and *Drc1*^W244X/W244X^ adult mice did not exhibit any significant hydrocephaly relative to WT controls. (**B** and **C**) Average numbers of pups per litter when crossing WT, *Drc1*^R554X/R554X^ and *Drc1*^W244X/W244X^ males with WT females, *n* = 3, *P* < 0.01. Data are represented as the mean ± SEM. (**D**) DRC1 was not detected in *Drc1*^R554X/R554X^ and *Drc1*^W244X/W244X^ testis samples by western blotting. (**E** and **F**) qRT-PCR analyses of Drc1 levels in WT, *Drc1*^R554X/R554X,^ and *Drc1*^W244X/W244X^ testis, *n* = 3, *P* < 0.001. Data are represented as the mean ± SEM. (**G**, **I**) Testis from WT, *Drc1*^R554X/R554X^ and *Drc1*^W244X/W244X^ adult mice and (**H**, **J**) average testis weight/body weight did not differ significantly between groups, *n* = 4. Data are represented as the mean ± SEM. (**K**–**M**) Sections of periodic acid Schiff-stained testis, with asterisks being used to denote the lumen of seminiferous tubules, indicating a lack of sperm flagella of normal length.

Neither patient harboring DRC1 stop-gain mutations identified in this study exhibited respiratory symptoms or evidence of hydrocephaly. While humans exhibit a complex genetic background, inbred C57BL/6 mice present with a simplified background that may influence the penetrance of particular mutations. We noted that the knockout of other PCD-related genes including *Pcdp1* (44), *Camsap3* (45) and *Cep290* (46) in mice was lethal in inbred strains, whereas survival rates rose when the underlying genetic background was altered. We therefore speculated that the observed lethality associated with our *Drc1* mutant lines may have been attributable to the underlying genetic background of these animals. We thus hybridized *Drc1* mutant mice onto the C57BL/6 × ICR background and found that this significantly ameliorated the post-natal survival of these animals (Table 3). Mice that survived to adulthood did not exhibit hydrocephaly or decreased size (Fig. 3A). The fertility of individual male mice from these different strains (*Drc1^+/+^*, *Drc1*^R554X/R554X^ and *Drc1*^W244X/W244X^) was assessed by housing these animals with *Drc1^+/+^* (WT) females and recording the number of offspring per litter. Both *Drc1*^R554X/R554X^ and *Drc1*^W244X/W244X^ males failed to sire any offspring despite copulation with females (Fig. 3B and C). No DRC1 protein expression was detected in the testes of *Drc1*^R554X/R554X^ and *Drc1*^W244X/W244X^ mice, with no truncated protein being evident therein (Fig. 3D). Levels of *Drc1* mRNA were also significantly lower in these tissue samples, consistent with an NMD phenotype associated with these mutations mRNA (Fig. 3E and F). No differences in gross testis appearance or weight were noted when comparing *Drc1^+/+^* and *Drc1*^R554X/R554X^ littermates (Fig. 3G and H), and the same was true when comparing *Drc1^+/+^* and *Drc1*^W244X/W244X^ samples (Fig. 3I and J). Following PAS staining, we found that both *Drc1*^R554X/R554X^ and *Drc1*^W244X/W244X^ lacked flagella of normal length within the lumen of seminiferous tubules (Fig. 3K–M). Decreased sperm counts and the presence of short or absent flagella were also observed in cauda epididymis sections from *Drc1*^R554X/R554X^ and *Drc1*^W244X/W244X^ mice (Supplementary Material, Fig. S5). *Drc1*^−/−^ testis (Supplementary Material, Fig. S6) and epididymis (Supplementary Material, Fig. S5) tissues exhibited phenotypes similar to those of mice harboring the two base mutations detailed above, and as such *Drc1*^R554X/R554X^ and *Drc1*^W244X/W244X^ mice were the focus of subsequent experiments.

Almost all spermatozoa collected from the epididymal cauda in *Drc1*^R554X/R554X^ and *Drc1*^W244X/W244X^ mice exhibited abnormalities including short, bent, curled, thick or absent flagella (Supplementary Material, Fig. S7). Immunofluorescent staining confirmed DRC1 to be completely absent in these *Drc1*^R554X/R554X^ and *Drc1*^W244X/W244X^ spermatozoa (Supplementary Material, Fig. S9). Normal sperm acrosome morphology was detected in *Drc*^R554X/R554X^ and *Drc1*^W244X/W244X^ mice, whereas sperm tails were clearly disordered (Supplementary Material, Fig. S8). These serious tail defects coincided with a complete absence of sperm motility for both murine strains (Supplementary Material, Movies S1–S3). Altogether, these findings indicate that DRC1 is essential for sperm flagellum formation and male fertility.

### DRC1 is an essential N-DRC assembly mediator in both flagella and cilia

**Figure 4:**
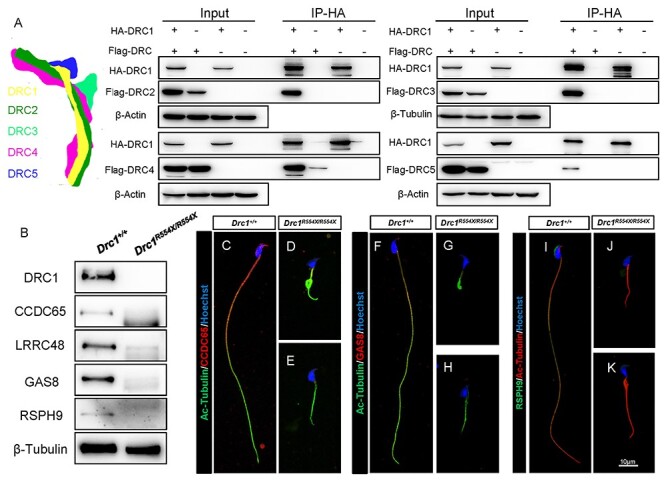
DRC1 is an essential component of N-DRC assembly in sperm flagella. (**A**) Individual DRC components were coexpressed in HEK293T cells. Interaction model of DRCs according to the structure of N-DRC in Chlamydomonas (11) (left) and immunoprecipitation of HA-DRC1 resulted in the co-precipitation of Flag-DRC2, Flag-DRC3, Flag-DRC4 and Flag-DRC5 (right). (**B**) Western blotting indicated that DRC1, CCDC65 (DRC2), LRRC48 (DRC3), GAS8 (DRC4) and RSPH9 could not be detected in mature *Drc1*^R554X/R554X^ sperm. (**C**–**E**) Immunofluorescence analysis of acetylated-tubulin (green) and CCDC65 (red) in WT and *Drc1*^R554X/R554X^ samples. (**F**–**H**) Immunofluorescence analysis of acetylated-tubulin (green) and GAS8 (red) in WT and *Drc1*^R554X/R554X^ samples. (**I**–**K**) Immunofluorescence analysis of acetylated-tubulin (red) and RSPH9 (green) in WT and *Drc1*^R554X/R554X^ samples.

**Figure 5:**
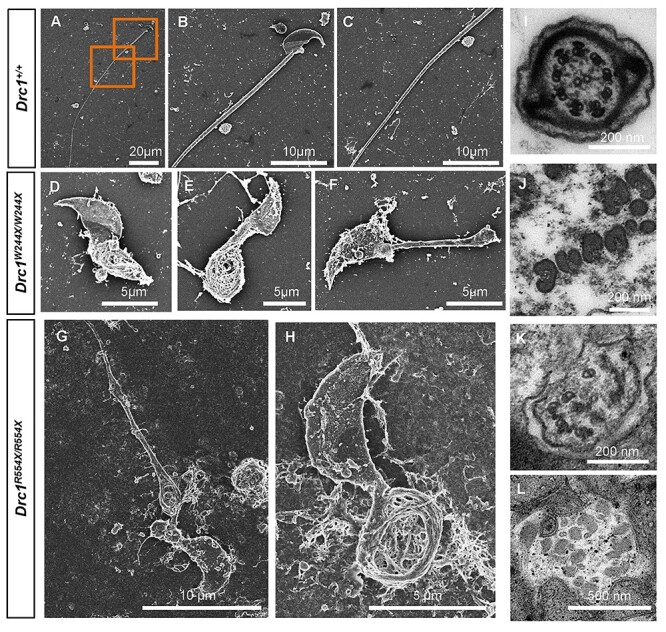
Sperm from Drc1 mutant mice exhibit distinct ultrastructural characteristics. (**A**–**C**) SEM revealed a mature sperm with a normal flagellum. (**D**–**H**) SEM revealed that the flagella of *Drc1*^W244X/W244X^ (**D**–**F**) and *Drc1*^R554X/R554X^ (**G** and **H**) sperm were distorted, exhibiting reticular, coiled and short phenotypes. (**I** and **J**) Transmission electron microscopy (TEM) was used to evaluate flagellar cross-sections for WT (I), *Drc1*^W244X/W244X^ (J) and *Drc1*^R554X/R554X^ (**K** and **L**) samples. The typical ‘9 + 2’ microtubule structure was evident for WT samples (I), while in *Drc1*^W244X/W244X^ (**J**) and *Drc1*^R554X/R554X^ (K and L) samples a disorganized axoneme with outer dense fibers separated by microtubules was evident, with an absence of N-DRC, RSs and dynein arms being evident.

We next conducted a co-IP experiment confirming the ability of DRC1 to interact with DRC2–5 (Fig. 4A). We then evaluated the expression of other DRC proteins in sperm to understand the impact of DRC1 knockout on N-DRC assembly. While β-Tubulin signals were largely unchanged, DRC1–4 could not be detected in *Drc1*^R554X/R554X^ mature sperm (Fig. 4B). The radial spoke (RS) component RSPH9 was also not detectable in these cells (Fig. 4B). Immunofluorescent staining similarly indicated that CCDC65 (DRC2), GAS8 (DRC4) and RSPH9 signals were largely absent in *Drc1*^R554X/R554X^ spermatozoa (Fig. 4C–K). The same was true for *Drc1*^W244X/W244X^ spermatozoa (Supplementary Material, Fig. S10). Scanning electron microscope (SEM) analyses revealed clear distortion of the flagella of *Drc1*^R554X/R554X^ and *Drc1*^W244X/W244X^ sperm (Fig. 5A–H). Transmission electron microscope (TEM) further indicated that *Drc1*^R554X/R554X^ and *Drc1*^W244X/W244X^ sperm exhibited flagellum appendices such as outer dense fibers separated by microtubules, whereas N-DRC, RSs and dynein arms were not detectable (Fig. 5I–L).

**Figure 6:**
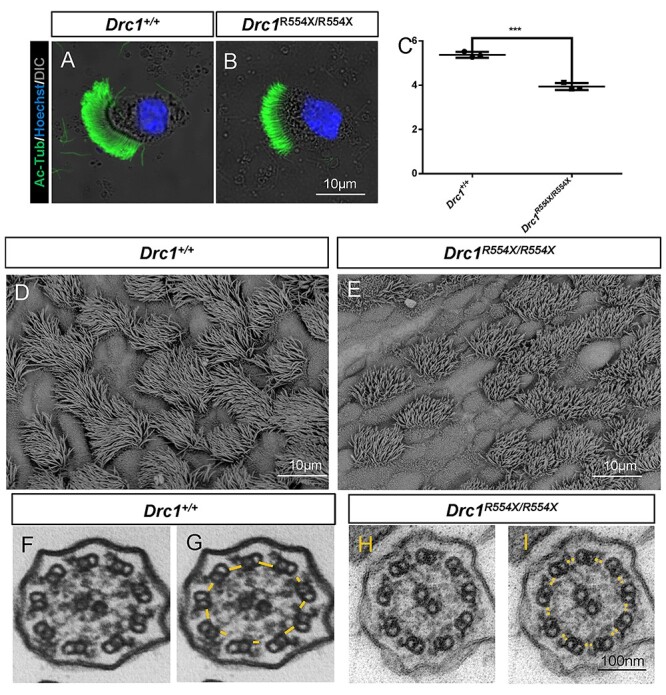
DRC1 is an essential component of N-DRC assembly in respiratory cilia. (**A** and **B**) Immunofluorescence analysis of acetylated-tubulin (green) in WT (A) and *Drc1*^R554X/R554X^ (B). (**C**) Analysis of cilia length in isolated respiratory cilia from WT and *Drc1*^R554X/R554X^ subjects. Each dot represents the average cilia length of one analyzed specimen (*n* = 3). Data are represented as the mean ± SEM. (**D** and **E**) SEM analyses of respiratory cilia from WT (D) and *Drc1*^R554X/R554X^ (E) subjects. (**F**–**I**) TEM was used to assess respiratory ciliary cross-sections for WT (F and G) and *Drc1*^R554X/R554X^ (H and I) subjects. The yellow solid line represents the N-DRC structure in WT samples, whereas it was not evident (yellow dotted line) in *Drc1*^R554X/R554X^ samples.

**Figure 7:**
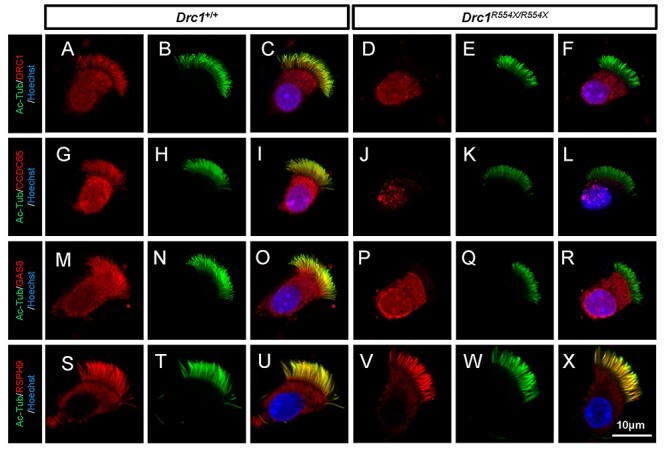
Immunofluorescence analysis of *Drc1^R554X/R554X^* respiratory cilia. (**A**–**R**) WT and *Drc1*^R554X/R554X^ respiratory epithelial cells were dual-stained with an antibody marker specific for the ciliary axoneme (acetylated-tubulin, green) and N-DRC antibody markers (DRC1, CCDC65 or GAS8, red). No N-DRC signal was evident in *Drc1*^R554X/R554X^ samples. (**S–X**) WT and *Drc1*^R554X/R554X^ respiratory epithelial cells were dual-stained with an antibody specific for the ciliary axoneme (acetylated-tubulin, green) and for the RS (RSPH9, red). RSPH9 signal did not differ between WT and *Drc1*^R554X/R554X^ samples.

We additionally assessed respiratory cilia changes in *Drc1*^R554X/R554X^ (Fig. 6) and *Drc1*^W244X/W244X^ mice (Supplementary Material, Fig. S12), revealing them to be significantly shortened (Fig. 6A–E, Supplementary Material, Fig. S12). While these respiratory cilia from *Drc1*^R554X/R554X^ and *Drc1*^W244X/W244X^ mice lacked N-DRC structure formation, they still maintained the 9 + 2 microtubule, RS and dynein arm structural arrangements in contrast to findings in sperm flagella (Fig. 6F–I). In B6 background, respiratory cilia were found no obvious different from mix background by light microscopy (Supplementary Material, Movie S7), while *TEM* analyses further indicated that the 9 + 2 microtubule was also maintained normally in *Drc1*^−/−^ (Supplementary Material, Fig. S11). Immunofluorescent staining confirmed the absence of DRC1, DRC2 and DRC4 in respiratory tract cilia from *Drc1*^R554X/R554X^ and *Drc1*^W244X/W244X^ mice (Fig. 7 and Supplementary Material, Fig. S13), whereas RSPH9 signal intensity was unchanged in these cilia, suggesting that the RS structure was unaffected by DRC1 deletion in respiratory tract cilia. Cilia lacking the N-DRC structure exhibited abnormal motility (Supplementary Material, Movies S4–S6). These data indicate that DRC1 is necessary for N-DRC complex assembly in both flagella and cilia.

### The loss of DRC1 disrupts flagellar axoneme assembly and causes nuclear deformation during spermiogenesis

**Figure 8:**
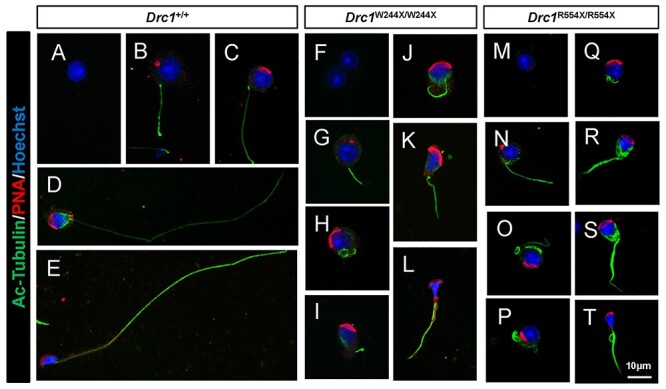
The absence of DRC1 results in a failure of flagellar axoneme assembly. (**A**–**T**) Immunofluorescence analysis of acetylated-tubulin (green) and PNA (red) from WT (A–E), *Drc1*^W244X/W244X^ (F–L) and *Drc1*^R554X/R554X^ (M–T) samples. (A–E) The axonemes were assembled normally to form a stable flagellar structure during spermiogenesis. (F–T) Axonemes were assembled normally in the early stage spermatids (G, N). Microtubule structures were disordered during spermatid differentiation (H–L, O–T).

While DRC1-deficient sperm exhibited disordered microtubular arrangement and RS formation, this was not observed in cilia. We therefore hypothesized that DRC1 plays a specific role in the context of sperm flagellum assembly or stabilization. To test this possibility, we examined flagellum formation during spermiogenesis (Fig. 8A–T), revealing that axoneme assembly occurred normally in early-stage spermatids from *Drc1*^R554X/R554X^ and *Drc1*^W244X/W244X^ mice (Fig. 8G and N), but that with spermatid differentiation, the microtubules within these cells became increasingly disordered such that a normal axoneme structure was no longer present (Fig. 8H–L and O–T). Flagellum lacking DRC1 appeared disordered, whereas the 9 + 2 microtubule structure was still evident in respiratory cilia from these animals, emphasizing that DRC1 plays a key role in regulating the structural stability of sperm flagellar axonemes.

Sperm from patients aIV-1 and bIV-1 exhibited a high frequency of head deformities in addition to the aforementioned tail deformities, and such head deformities were also common in sperm from *Drc1*^R554X/R554X^ and *Drc1*^W244X/W244X^ mice (Supplementary Material, Fig. S8). This may be attributable to the microtubular structural disorder observed in the cytoplasm of spermatids during spermiogenesis. When we further analyzed dynamic changes in the perinuclear manchette microtubule structure during spermiogenesis, we observed a long and narrow manchette distribution around the nucleus in the sperm of *Drc1*^R554X/R554X^ and *Drc1*^W244X/W244X^ mice (Supplementary Material, Fig. S14A–O), resulting in the amorphous head phenotypes of these cells. Overall, these data suggest that sperm flagellar microtubule disorder as a consequence of DRC1 deletion can additionally impact dynamic cytoplasmic microtubule changes in spermatids, thereby causing complex head and tail defects.

## Discussion

PCD is a multisystem disease that typically arises due to mutations in abnormal genes associated with ciliary movement, with dynein arms, RS, N-DRC and related motility-associated structural defects having been linked to this condition in humans. PCD patients often exhibit a range of genetic defects in genes such as *DNAH1* (22), *CCDC114* (47) and *RSPH4A* (48), but phenotypic differences still exist even among individuals that share a given mutation. For example, *CEP290* mutations can cause a spectrum of phenotypes that range from retinal degeneration (Leber congenital amaurosis) to embryonic lethality (Meckel–Gruber syndrome) (49–55), and there are often no clear correlations between patient genotype and phenotype (46). Studies of knockout mice have similarly shown that the phenotypes of mice harboring mutations in different PCD-related genes vary depending on the parental background strain (44–46,56). In the present report, we detected no respiratory symptoms harboring *DRC1* mutations, but we did identify a previously unreported MMAF phenotype in these patients. To confirm the relationship between *DRC1* mutations and these phenotypes, we generated three *Drc1* mutant mouse strains. The phenotypic manifestations of this mutation were dependent upon murine genetic background such that *Drc1^−/−^* and *Drc1*^R554X/R554X^ mice on the C57BL/6 background exhibited postnatal death. To determine whether this mutation was mutation-specific, we also assessed an additional *DRC1* mutation recorded in gnomAD predicted to cause premature stop codon generation. Prepubertal death was also observed for *Drc1*^W244X/W244X^ mice on the C57BL/6 background and as such we not able to use these mice to model male infertility. When we instead introduced the ICR background into these *Drc1^−/−^*, *Drc1*^R554X/R554X^ and *Drc1*^W244X/W244X^ strains, we found that some of these mice survived and exhibited weight and behaviors comparable to those of WT control mice. Importantly, these mice recapitulated the MMAF phenotypes detected in humans with a *DRC1* deficiency. These results confirmed the ability of *DRC1* mutations to directly cause MMAF while also reaffirming the fact that genetic background is a key determinant of PCD pathogenesis. While we did not explore the genomic basis for this finding, it nonetheless underscores the contribution of individual genetic background to PCD patient phenotypes.

Few prior studies have explored spermiogenesis in the context of MMAF pathogenesis. We found that sperm flagellum damage was more severe than ciliary damage in the present study, as evidenced by axoneme structural disorder in addition to DRC structural loss. During the early stages of spermiogenesis, axonemes appeared similar in *Drc1^+/+^*, *Drc1*^R554X/R554X^ and *Drc1*^W244X/W244X^ round spermatids. However, axoneme structural disorder was evident in *Drc1*^R554X/R554X^ and *Drc1*^W244X/W244X^ elongating spermatids and spermatozoa, and these phenotypic changes cannot be explained by the known functional role of DRC, suggesting that DRC1 to additionally be necessary for flagellar structural stability. We simultaneously detected manchette structural abnormalities in elongating spermatids from mice harboring these DRC1 mutations, further contributing to sperm nuclear transformation and disorder. Similar findings have also been observed in mice exhibiting sperm flagellar dysplasia including *Drc7*^−/−^, *Rsph6a*^−/−^ and *Cfap43*^−/−^ animals (15,57,58). These data suggest that abnormal microtubule organization in sperm flagella is associated with abnormal microtubule organization in the elongating spermatid manchette, potentially explaining the mixed head and tail malformations observed in the context of teratospermia.

**Figure 9:**
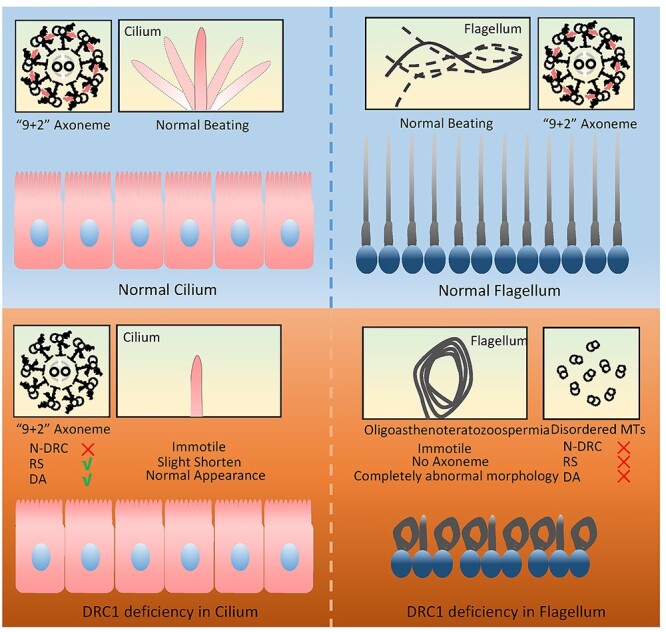
A model of distinct phenotypic manifestations in cilia and flagella of DRC1 deficiency.

In summary, we herein identified a novel *DRC1* mutation that causes MMAF and male infertility, and we found that genetic background profoundly influences the phenotypic manifestation of this mutation. These data offer new insights regarding the genetic basis for phenotypic diversity in PCD patients, emphasizing the fact that differences in genetic background can influence the penetrance of different PCD-related mutations. We also found that DRC1 mutations resulted in decreased flagellum axoneme stability, thereby causing flagellar structural disorder while also affecting the manchette of spermatids. These DRC1 mutations also led to distinct phenotypic manifestations in cilia and flagella, providing a foundation for the future study of flagellar and ciliary structural stability (Fig. 9). Overall, our data emphasize the important functions of the N-DRC core component DRC1 in mammals.

## Materials and Methods

### Study participants

A cohort of 100 Han Chinese men with MMAF-related male infertility were enrolled from the First Affiliated Hospital of Anhui Medical University and the Affiliated Suzhou Hospital of Nanjing Medical University in China. All enrolled patients exhibited primary infertility, and patients with PCD were excluded. To be eligible for enrollment, patients had to present with a standard MMAF phenotype characterized by severe asthenozoospermia (total sperm motility < 10%; normal: 40%) with >40% of spermatozoa exhibiting the following flagella abnormality: short, absent, coiled, bent or irregular flagella. Karyotypic analyses for all enrolled patients were normal (46, XY), as were hormone levels, bilateral testicular size distributions and secondary sex characteristics. The samples of parental DNA were obtained from 84 of the 100 enrolled MMAF patients. An EX20 kit (AGCU ScienTech Incorporation, Wuxi, China) was used to confirm parental relationships for the enrolled patients. The Institutional review boards of the School of Basic Medical Sciences, Nanjing Medical University, School of Life Sciences at Fudan University, the First Affiliated Hospital of Anhui Medical University and the Affiliated Suzhou Hospital of Nanjing Medical University approved this study. All patients provided informed consent to participate, and the study was performed as per the Declaration of Helsinki.

### Whole-exome sequencing

Whole-exome sequencing (WES) was conducted for the 100 enrolled MMAF patients using gDNA isolated from peripheral blood with a DNeasy Blood and Tissue kit (QIAGEN, Duesseldorf, Germany). An Agilent SureSelectXT Human All Exon Kit was utilized to isolate and enrich exonic sequences, after which sequencing was performed on the Illumina HiSeq X-TEN platform. Standard assembly (Burrows-Wheeler Aligner), calling (Genome Analysis Toolkit) and annotation (ANNOVAR) were performed for sequencing analyses as detailed previously (37), and DRC1 mutations identified via this approach were confirmed via Sanger sequencing using primers listed in Supplementary Material, Table S1.

### Animals

Mice were housed in a standard animal facility (20–22°C; 50–70% humidity; 12 h light/dark cycle) with free food and water access. The Institutional Animal Care and Use Committees of Nanjing Medical University approved this study (Approval No. IACUC-1810020), and all experiments were performed as per the Guide for the Care and Use of Laboratory Animals and institutional guidelines.

### qPCR

Trizol (Thermo Fisher, Waltham, MA, USA) was used to extract RNA samples, after which 1 μg of total RNA was used to prepare cDNA with a HiScriptIII RT SuperMix (Vazyme, R323, Nanjing, China) as per the manufacturer’s instructions. These cDNA samples were then diluted 1:4 and analyzed via qPCR in a 20 μl volume containing 250 nmol/l of each appropriate primer, 1 μl of cDNA and AceQ qPCR SYBR Green Master Mix (Vazyme, Q131, Nanjing, China). Thermocycler settings were as follows: 50°C for 2 min; 95°C for 5 min; 40 cycles of 95°C for 10 s and 60°C for 30 s. The 18 s rRNA was utilized as a normalization control, and all primer sequences are compiled in Supplementary Material, Table S2.

### Antibodies

Rabbit anti-RSPH9 (23253-1-AP) and anti-CENTRIN1 (12794-1-AP) were obtained from Proteintech (Wuhan, China). Rabbit anti-β-Actin (AC026) and mouse anti-β-Tubulin (AC021) were from Abclonal (Wuhan, China). Rabbit anti-Acetylated Tubulin was purchased from Cell Signaling Technology (MA, USA). Mouse anti-FLAG M2 (F3165), anti-Acetylated Tubulin (T6793) and anti-α-Tubulin (T9026) were from Sigma-Aldrich (St. Louis, MO,USA). Mouse anti-HA-tag (M180–3) and mouse anti-DDDDK-tag (PM020) were from Medical & Biological Laboratories (Nagoya, JP).

Antibodies specific for DRC1, CCDC65, LRRC48 and GAS8 were prepared as per previously published protocols (59). Briefly, murine DRC1 (aa 1–146), CCDC65 (aa 1–126), LRRC48 (aa 182–300) and GAS8 (aa 1–478) were expressed as His fusion proteins in *Escherichia coli* using the pET-28a (+) vector, after which the Ni-NTA His Bind Resin was used to affinity purify these proteins. For mice were then immunized with the resultant fusion proteins, yielding the four following antiserum preparations: anti-DRC1, anti-CCDC65, anti-LRRC48 and anti-GAS8.

### *Drc1* mutant mouse generation

*Drc1^−/−^* and *Drc1*^R554X/R554X^ mice were prepared via CRISPR/Cas9 genome editing, while *Drc1*^W244X/W244X^ mice were generated using Cytosine base editors and BE3. For Drc1^R554X/R554X^ and Drc1 knock-out mice, appropriate PM sgRNA and donor target sequences were selected to achieve the nonsense mutations and consequent deletion of *Drc1* exon 13. For *Drc1*^W244X/W244X^, the BE3 sgRNA was chosen to introduce a *Drc1* nonsense mutation. The PM sgRNA, BE3 sgRNA and Donor sequences used herein were 5′-GACGGTGGGCTCGGTATCGAAGG-3′, 5′-CTCCCATTTCTTCTTATTGCTGG-3′ and 5′-CTTATACAAGCTGGTAAACTTCTTCCTCCGATACTGAGCCCACCGTCTATCCTCTGCCCAGGTGAGACGGGCAGCTGGGGAGAGCATTTCCTTTCGTTC-3′, respectively. The two complementary DNA oligos of these sgRNAs were separately annealed and ligated to the BsaI-digested pUC57-T7-sgRNA vector. The sgRNA templates were obtained from sgRNA plasmids by PCR amplification with Trans PCR F (5′-GAAATTAATACGACTCACTATAGG-3′) and Trans PCR R (5′-AAAAGCACCGACTCGGTGCCA-3′) primers. A MinElute PCR Purification Kit (28004, QIAGEN, Duesseldorf, Germany) was then used to purify these PCR products. A MEGAshortscript Kit (AM1354, Ambion, Austin, TX, USA) was used for sgRNA preparation, followed by purification based on directions provided with the MEGAclear Kit (AM1908, Ambion, Austin, TX, USA). Cas9 (Addgene No. 44758) and BE3 (Addgene No. 73021) plasmids were linearized using AgeI and PmeI and were purified with the MinElute PCR Purification Kit (28004, QIAGEN, Duesseldorf, Germany). BE3 and Cas9 mRNA were generated via *in vitro* transcription with the mMESSAGE mMACHINE T7 Ultra Kit (AM1345, Ambion, Austin, TX, USA), after which an RNeasy Mini Kit (74104, QIAGEN, Duesseldorf, Germany) was used for purification based upon provided directions. One group of murine zygotes were coinjected with Cas9 mRNA (50 ng/μl), PM sgRNA (20 ng/μl) and Donor (50 ng/ul), while another group was coinjected with BE3 mRNA (50 ng/μl) and BE3 sgRNA (20 ng/μl). After injection, these zygotes were transferred into pseudo-pregnant recipients. At 7 days of age, toe-cutting was used to tag newborn mice, and DNA from these excised tissue samples was assessed with the Mouse Direct PCR Kit (B40013, Biotool, Houston, TX,USA). PCR was conducted using model-appropriate primers (*Drc1^−/−^* and *Drc1^R554X/R554X^* mice: F 5′-TTGGTGTCATGTTCTGTGT-3′, R 5′-CTATAAGCCGATGGTATTAGC-3′; *Drc1^W244X/W244X^* mice: F 5′-GCAGTTATGAAGTAGCAAGT-3′, R 5′-GGTCGTCCTGAACATAGAA-3′) with the PrimeSTAR HS DNA Polymerase (DR010A, Takara, Tokyo, Japan) and the following thermocycler conditions: 95°C for 5 min; 35 cycles of 95°C for 30 s, 62°C (−0.2°/Cycle) for 30 s and 72°C for 30 s; 72°C for 5 min. Sanger sequencing of PCR products was then performed.

### Fertility test

Fertility tests were conducted for adult mice of each genotype. Males were mated with three WT ICR mice, with vaginal plug inspections being conducted every morning. Dates of birth and number of pups per litter were recorded.

### Sperm analysis

Sperm were collected from the cauda epididymis, extruded and suspended in modified HTF Medium (Irvine Scientific, CA, USA) containing 10% FBS. After a 5 min incubation at 37°C, sperm samples (10 μl) were subjected to computer-assisted semen analysis (Hamilton-Thorne Research, Inc., MA, USA). Remaining sperm samples were fixed for 30 min with and spread onto slides, after which H&E staining was performed as per standard methods to assess sperm morphology.

### Histological analysis

Mouse testes, epididymal and tracheal tissues were collected from a minimum of three mice per genotype. Modified Davidson’s fluid was used to fix testis and epididymis samples for up to 24 h, whereas 4% PFA was used to fix tracheal samples overnight followed by storage in 70% ethanol. Samples were then dehydrated via ethanol gradient, paraffin-embedded and 5 μm thick tissue sections were mounted on glass slides. H&E staining was conducted as per standard protocols while Periodic Acid-Schiff (PAS) staining was conducted with the PAS staining kit (395B, Sigma-Aldrich, St. Louis, MO, USA).

### Murine tracheal epithelial cell isolation

A tracheal brushing approach was used to isolate murine tracheal epithelial cell (mTECs) as in prior studies (60). Briefly, tracheal brushing was conducted to isolate multiciliated airway cells, which were fixed for 30 min with 4% PFA, spread on glass slides and allowed to air-dry.

### Western blotting

A lysis buffer (50 mM Tris–HCl pH 8.2, 75 Mm NaCl, 8 M urea) containing a 1× cOmplete™ EDTA-free Protease Inhibitor Cocktail (Roche, Basel, Switzerland) was used to extract proteins, which were then separated via SDS-PAGE and transferred onto PVDF membranes that were blocked for 2 h with 5% non-fat milk in TBS at room temperature, followed by overnight incubation with appropriate primary antibodies at 4°C. Blots were then washed thrice with TBST, probed for 2 h at room temperature with appropriate secondary antibodies and protein bands were then detected with the high-sig ECL western blotting substrate (Tanon, Shanghai, China).

### Cell culture

HEK293T cells were grown in high-glucose DMEM containing 10% FBS (Gibco, Grand Island, NY,USA) and penicillin/streptomycin (100 U/ml, Thermo Fisher, Waltham, MA, USA). Lipofectamine 2000 (11 668 019, Thermo Fisher) was used for cellular transfection based upon provided directions.

### Immunoprecipitation

Lipofectamine 2000 was used to transfect HEK293T cells with DRC expression plasmids. At 2 days post-transfection, cells were lysed for 40 min using RIPA buffer (P0013C, Beyotime, Shanghai, China) containing 1× cOmplete EDTA-free Protease Inhibitor Cocktail (Roche, Basel, Switzerland) at 4°C, after which samples were spun down for 20 min at 12000 × g. Supernatants from these lysates were then precleared for 1 h with 10 μl Protein G magnetic beads (10004D, ThermoFisher, Waltham, MA, USA) at 4°C, after which they were combined with anti-HA-tag antibodies overnight at 4°C. They were then mixed with 50 μl of Protein G magnetic beads for 3 h at 4°C. Beads were washed thrice with RIPA buffer, boiled for 10 min in 1× SDS loading buffer and proteins were then subjected to SDS-PAGE analysis.

### Immunofluorescence

Immunofluorescent staining of testis and tracheal tissue sections was conducted as detailed previously (61). Spermatozoa and mTECs were obtained as detailed above. Samples were washed thrice with PBS (10 min/wash), and antigen retrieval was conducted for 10 min in 10 mM citrate buffer (pH 6.0) in a microwave oven. Following three additional PBS washes, 5% BSA was used to block slides for 2 h, after which they were stained overnight with appropriate primary antibodies at 4°C. Following secondary antibody staining for 2 h at room temperature, Hoechst 33342 counterstaining was performed for 5 min. Slides were then rinsed with PBS and mounted using glycerol prior to imaging with an LSM800 confocal microscope (Carl Zeiss AG, Jena, Germany) or a TCS SP8X confocal microscope (Leica Microsystems, Wetzlar, Germany).

### Analysis of tracheal ciliary length

Tracheas were excised from three 12-day-old *Drc1*^+/+^, *Drc1*^W244X/W244X^ and *Drc1*^R554X/R554X^ mice, and isolated mTECs were stained with anti-acetylated α-tubulin as above. Cells were then imaged via confocal microscope (Leica TCS SP8X), and LAS X was used to measure cilia length by assessing the ciliary tuft length for each cell, with 20 cells being analyzed per animal.

### Assessment of tracheal ciliary motility

Murine tracheal tissues were dissected, added to high-glucose DMEM containing 10% FBS (Gibco, Grand Island, NY,USA), opened on the dorsal side and minced under stereoscopic magnification to yield ~5 mm tissue fragments. These tissues were then transferred to a confocal dish (BDD012035, BIOFIL, Guangzhou, China) and a scotch tape spacer was used to facilitate their imaging under a 40× objective (CFI S Plan Flour ELWD NAMC) with an inverted microscope (Eclipse Ti2-U, Nikon, Tokyo, Japan).

### Transmission electron microscopy

For ultrastructural analyses, 2.5% glutaraldehyde was used to fix tracheal and epididymal tissue samples overnight followed by 2% OsO4 post-fixing and embedding in Araldite. Ultrathin 80 nm sections were then stained using uranyl acetate and lead citrate, followed by analysis with an electron microscope (JEM.1010, JEOL, Tokyo, Japan).

### Scanning electron microscopy

Spermatozoa and tracheal samples were fixed for 2 h with 2.5% phosphate-buffered glutaraldehyde at 4°C. Spermatazoa were then allowed to attach to coverslips coated with poly-L-lysine. Both sample types were then washed with PBS, dehydrated with a chilled ethanol gradient (30, 50, 70, 80, 90 and 100%) and subjected to critical point drying with a Lecia EM CPD300 Critical Point Dryer (Wetzlar, Germany). Samples were then attached to appropriate specimen holders and coated with gold particles via the use of an ion sputter coater (EM ACE200, Leica, Wetzlar, Germany). A Helios G4 CX scanning electron microscopy (SEM) (Thermo Scientific, Waltham, MA, USA) was then used to image samples.

### Statistical analysis

Experiments were conducted in triplicate. Data are given as means ± standard error and were compared by one-way ANOVAs and unpaired two-tailed *t*-tests. *P* < 0.05 was the significance threshold. Microsoft Excel and GraphPad Prism 6.0 were utilized for all statistical testing.

## Web Resources

The URLs for data presented herein are as follows:

1000 Genomes Project, http://www.internationalgenome.org

gnomAD, http://gnomad.broadinstitute.org

GTEx, http://www.gtexportal.org

Human Protein Atlas, https://www.proteinatlas.org

NCBI, https://www.ncbi.nlm.nih.gov/

OMIM, http://www.omim.org

Picard, https://github.com/broadinstitute/picard

PolyPhen-2, http://genetics.bwh.harvard.edu/pph2/

SIFT, https://sift.bii.a-star.edu.sg

UCSC Genome Browser, http://genome.ucsc.edu

## Data and code availability

The NCBI reference sequence numbers for human *DRC1* transcript, DRC1, mouse *Drc1* transcript and DRC1 are GenBank: NM_145038.5, NP_659475.2, NM_001033460.4 and NP_001028632.1, respectively.

## Supplementary Material

Movie_S1_ddab171

Movie_S2_ddab171

Movie_S3_ddab171

Movie_S4_ddab171

Movie_S5_ddab171

Movie_S6_ddab171

Movie_S7_ddab171

Supporting_Information_ddab171
