# Supplementary material for: Loss of DRC1 function leads to multiple morphological abnormalities of the sperm flagella and male infertility in human and mouse
Source: Hum Mol Genet. 2021 Jun 24;30(21):1996–2011. doi: 10.1093/hmg/ddab171 (PMC8522639; doi:10.1093/hmg/ddab171)
Supplement: Supporting_Information_ddab171 [file supporting_information_ddab171.zip › Supporting_Information_ddab171.docx]

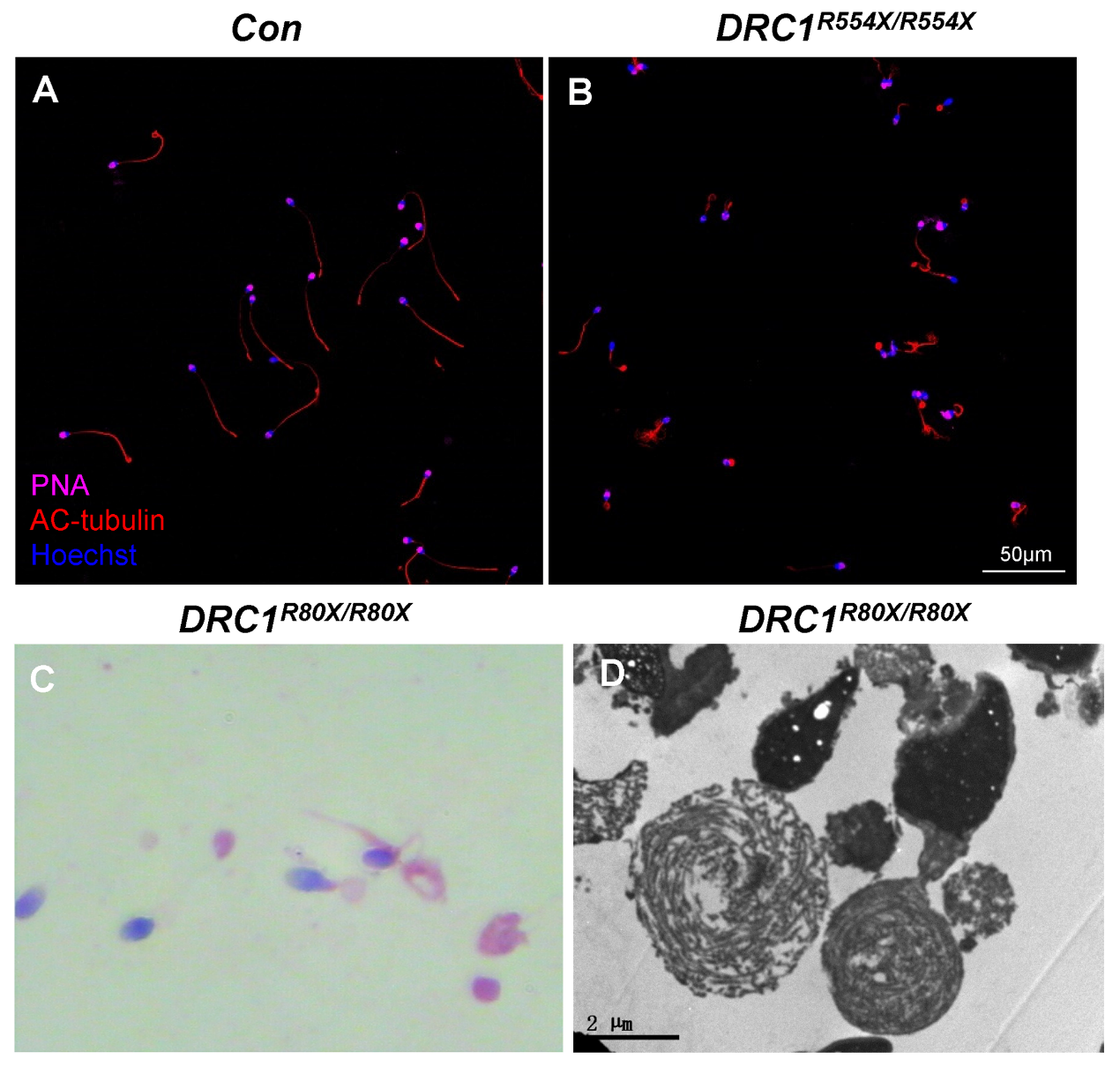


**Figure S1**

**The *DRC1*^R80X/R80X^ mutation causes multiple morphological abnormalities and ultrastructural disorders in sperm flagella**

(A-B) Spermatozoa from a fertile control individual (A) and from a *DRC1*^R554X/R554X^ mutant individual (B) were stained with anti-Centrin-1, anti-Ac-Tubulin, and PNA. (C) Most spermatozoa from *DRC1*^R80X/R80X^ probands exhibited flagellar morphological abnormalities. (D) TEM revealed abnormal sperm flagellar structures in *DRC1*^R80X/R80X^ probands.


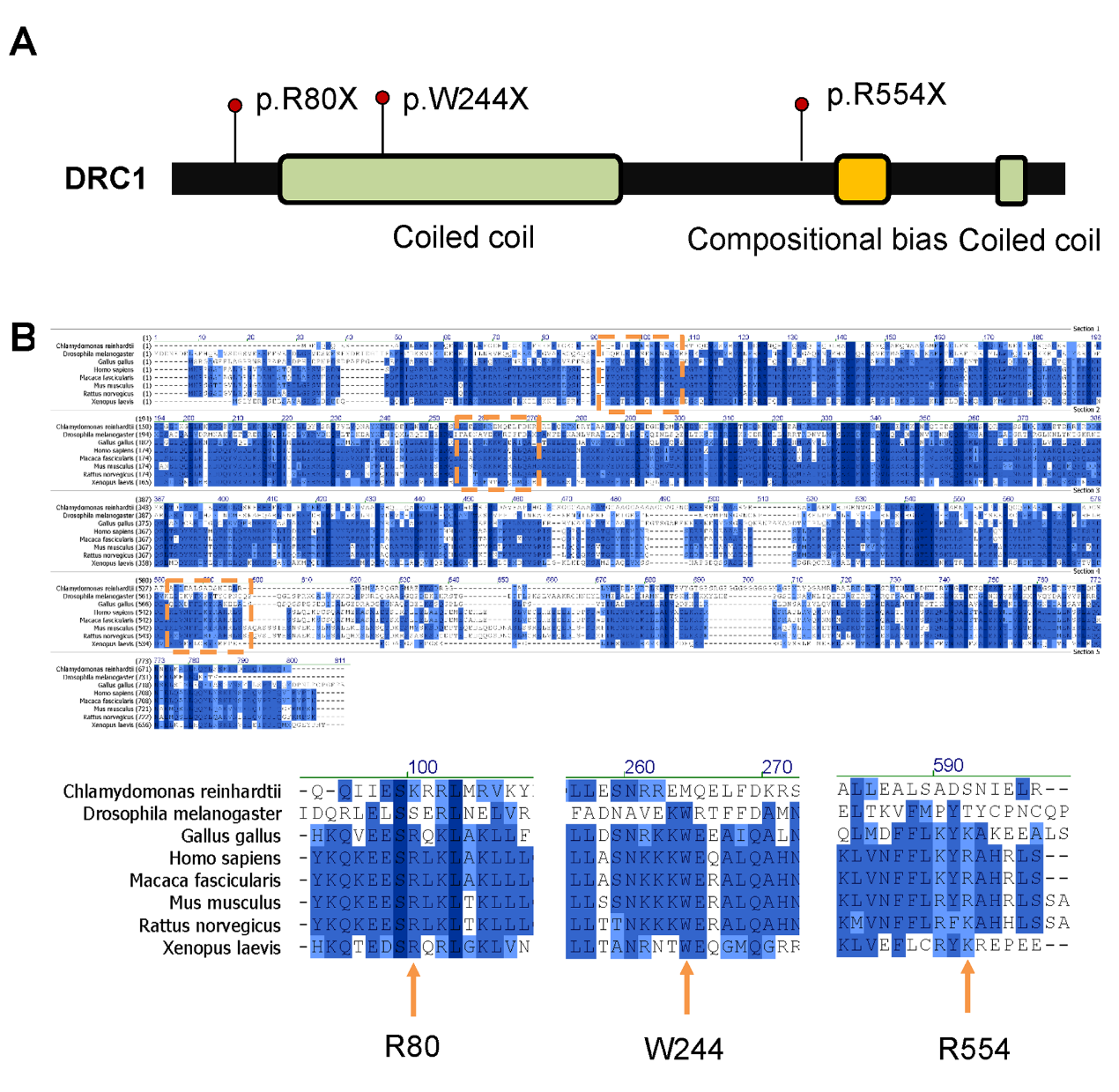


**Figure S2**

**Conservation analysis of DRC1 in various species**

(A) The protein structure of DRC1 and its mutant positions (R80, W244, R554). (B) DRC1 protein sequence similarity in various species (upper), and an enlarged view of mutant sites (lower). A dark blue background indicates identical residues in all species while a light blue background indicates weakly similar residues.


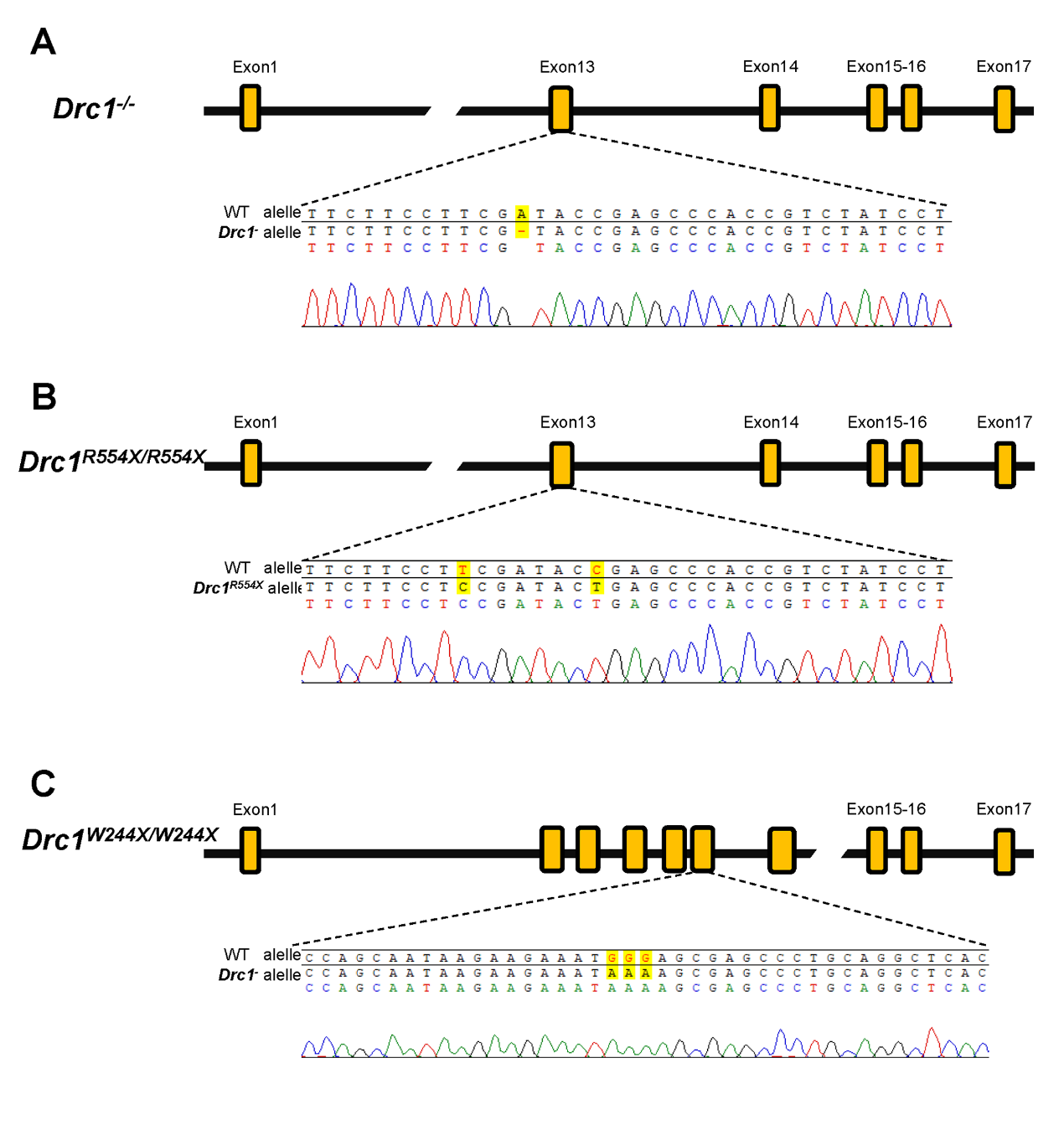


**Figure S3**

**CRISPR/Cas9 targeting strategy overview**

(A)*Drc1* mutant mice carried a 1-bp deletion within exon13 of this gene, and were referred to as *Drc1^-/-^* animals. (B) The murine model of the R554 mutation in MMAF patients was obtained via a C-to-T mutation resulting in a stop-gain (*Drc1*^R554X/R554X^). (C) The editing strategy for the W244 mutation resulted in the mutation of a TGGG sequence to TAAA, resulting in a stop-gain (*Drc1*^W244X/W244X^).


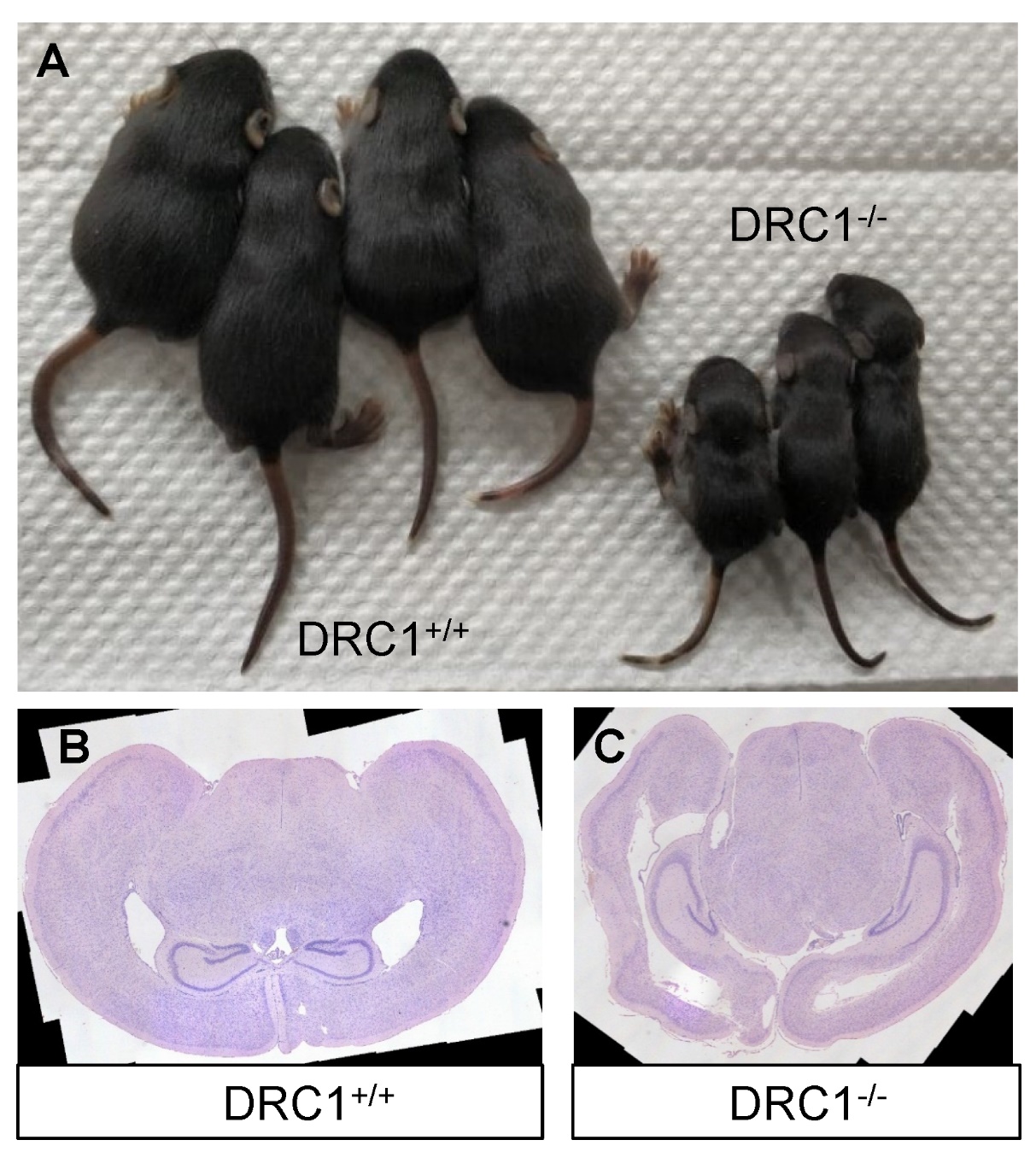


**Figure S4**

**DRC1 knockout mice on the C57BL/6 background suffer from hydrocephaly and postnatal death**

(A) Drc1^-/-^ mice were significantly smaller than *Drc1*^+/+^ mice at 14 days after birth, and exhibited obvious hydrocephalus. (B-C) H&E stained coronal sections of P14 *Drc1*^+/+^ and *Drc1*^-/-^ brain tissues.


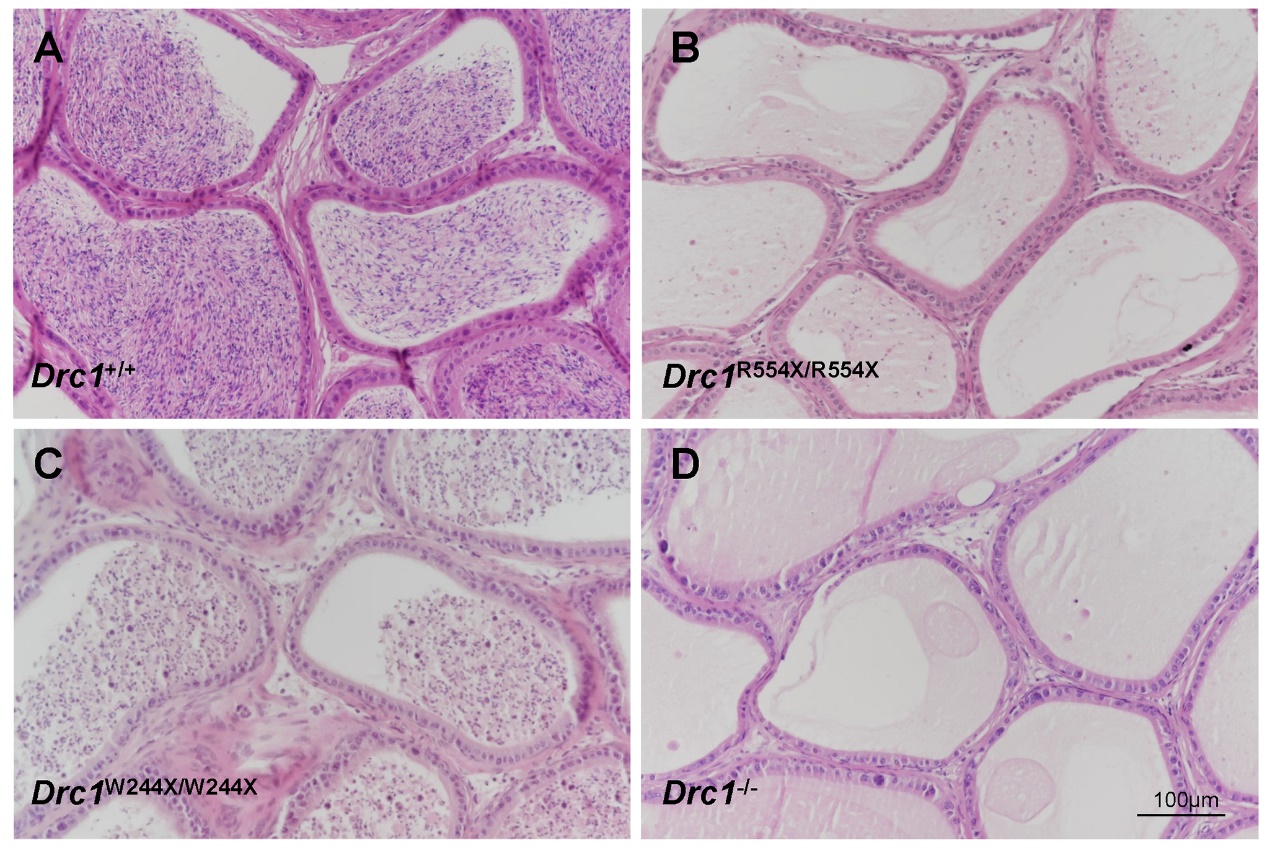


**Figure S5**

**Morphology of cauda epididymis sections**

(A-D) Sections of hematoxylin and eosin-stained cauda epididymis from wild-type (A), *Drc1*^R554X/R554X^ (B), *Drc1*^W244X/W244X^ (C), and *Drc1*^-/-^ (D) subjects.


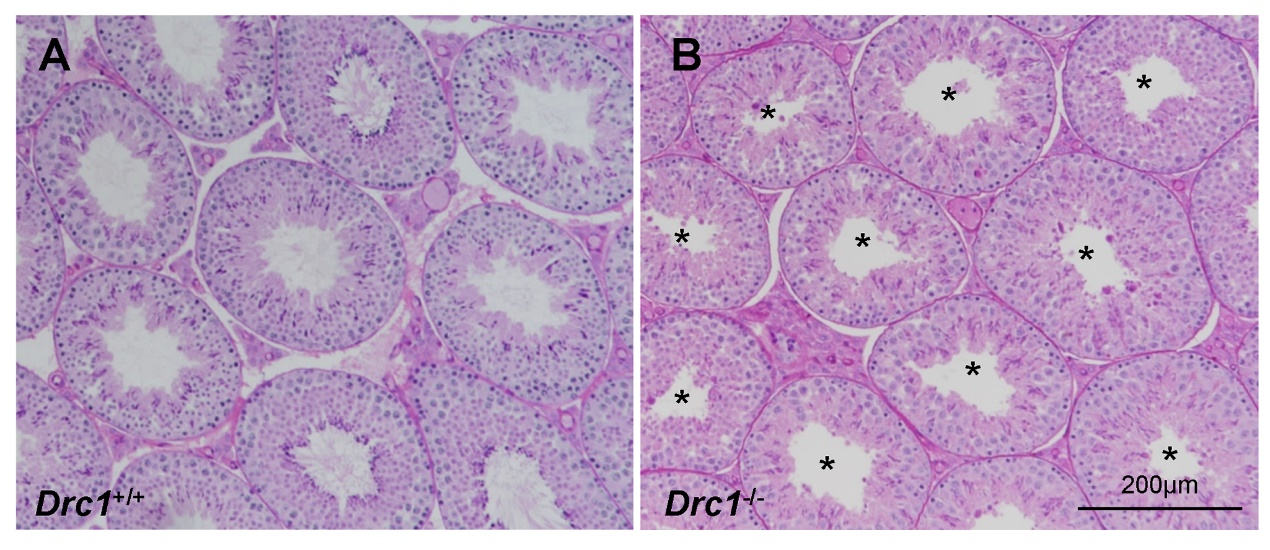


**Figure S6**

**Analyses of testis morphology**

(A-B) Sections of PAS-stained cauda epididymis from wild-type (A) and *Drc1^-/-^*(B) subjects.


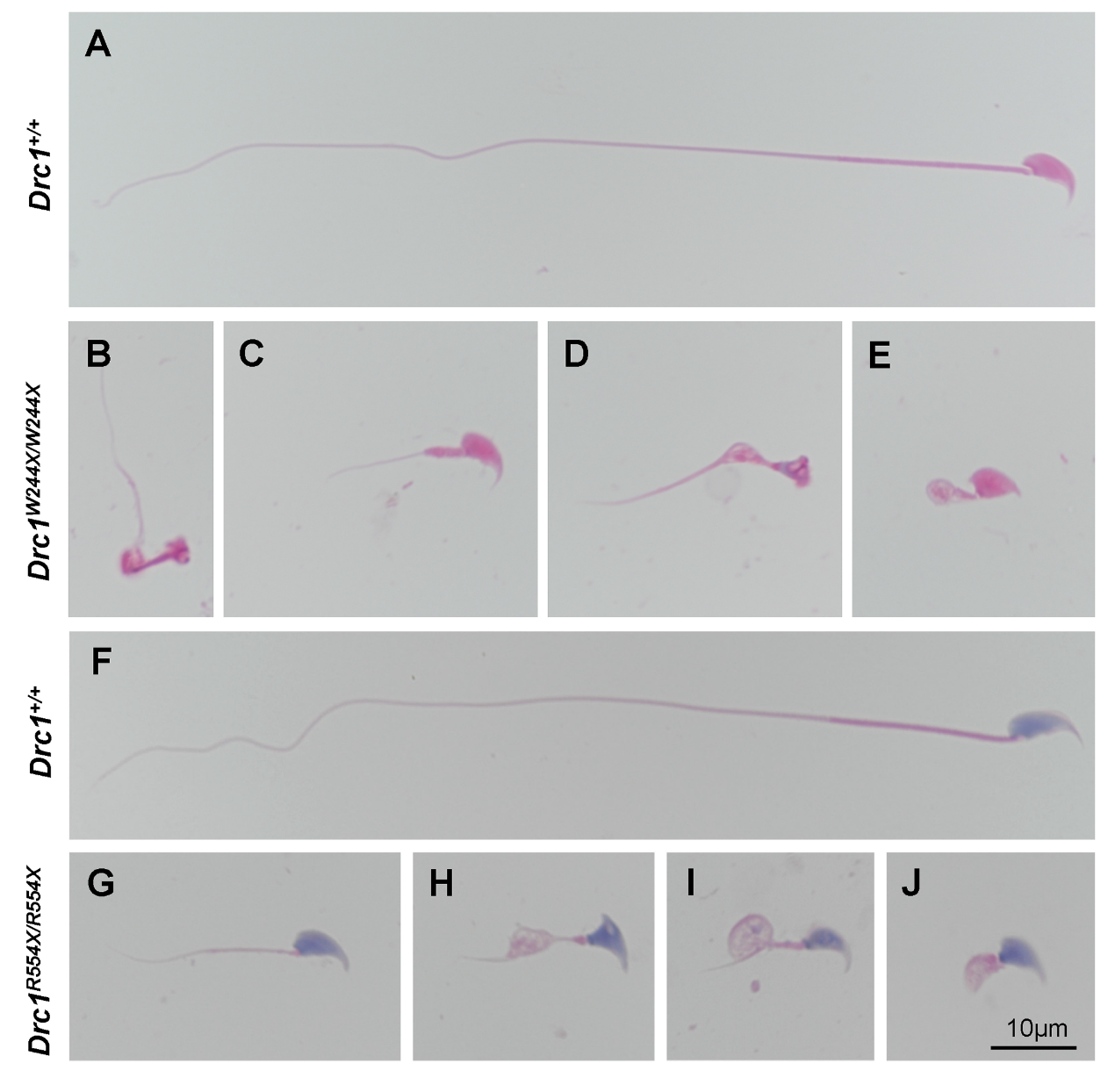


**Figure S7**

**Assessment of *Drc1*^W244X/W244X^ and *Drc1*^R554X/ R554X^ sperm morphology in male mice.**

(A-J) Hematoxylin and eosin staining was used to analyze sperm from wild-type (A, F), *Drc1*^W244X/W244X^ (B-E) and *Drc1*^R554X/R554X^ (G-J) mice. Wild-type spermatozoa exhibited normal flagellar morphology, whereas *Drc1*^W244X/W244X^ and *Drc1*^R554X/R554X^ spermatozoa exhibited short, coiled flagella, or other MMAF phenotypes.


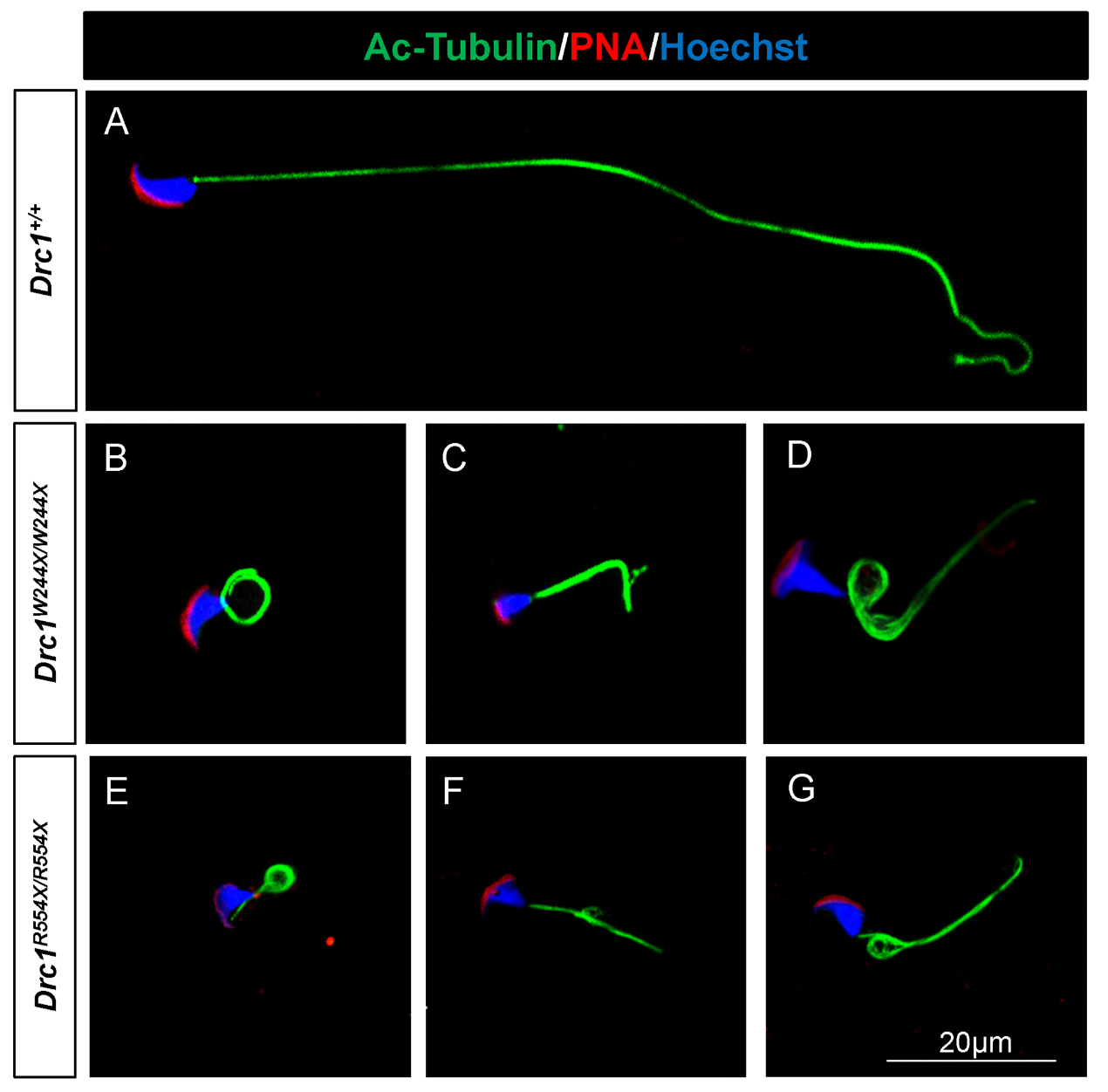


**Figure S8**

**Immunofluorescence analysis of spermatozoa**

Spermatozoa from wild-type (A), *Drc1*^W244X/W244X^ (B-D), and *Drc1*^R554X/R554X^ (E-G) male mice were stained with anti-Ac-Tubulin and PNA. Wild-type spermatozoa exhibited normal flagellar morphology, while *Drc1*^W244X/W244X^ and *Drc1*^R554X/R554X^ spermatozoa exhibited short, coiled flagella, or other MMAF phenotypes.


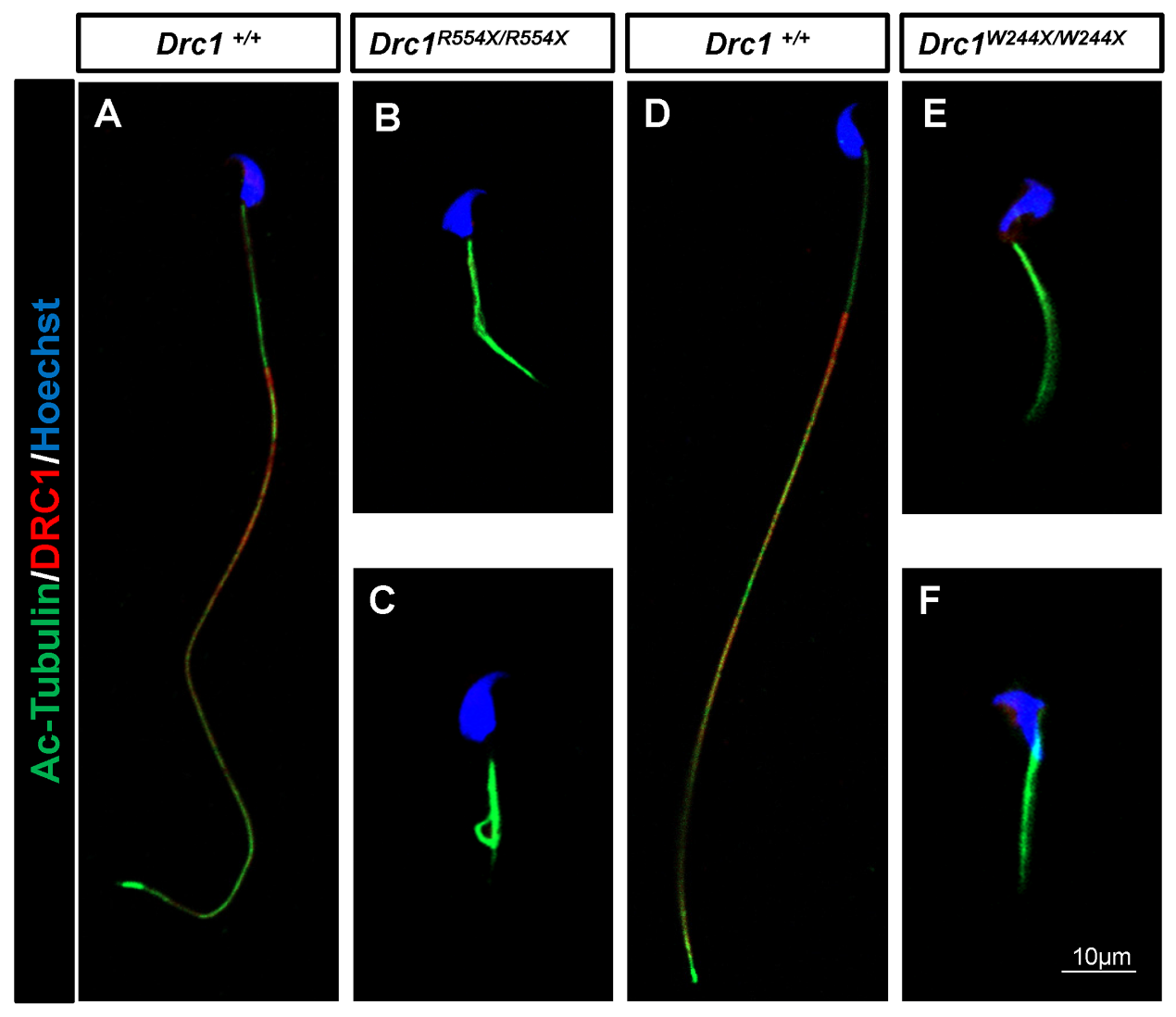


**Figure S9**

**DRC1 is absent in spermatozoa of *Drc1*^R554X/R554X^ and *Drc1*^W244X/W244X^ subjects.**

(A-F) Spermatozoa from wild-type (A, D), *Drc1*^R554X/R554X^ (B-C), and *Drc1*^W244X/W244X^ (E-F) subjects were stained with anti-DRC1 and anti-Ac-Tubulin.


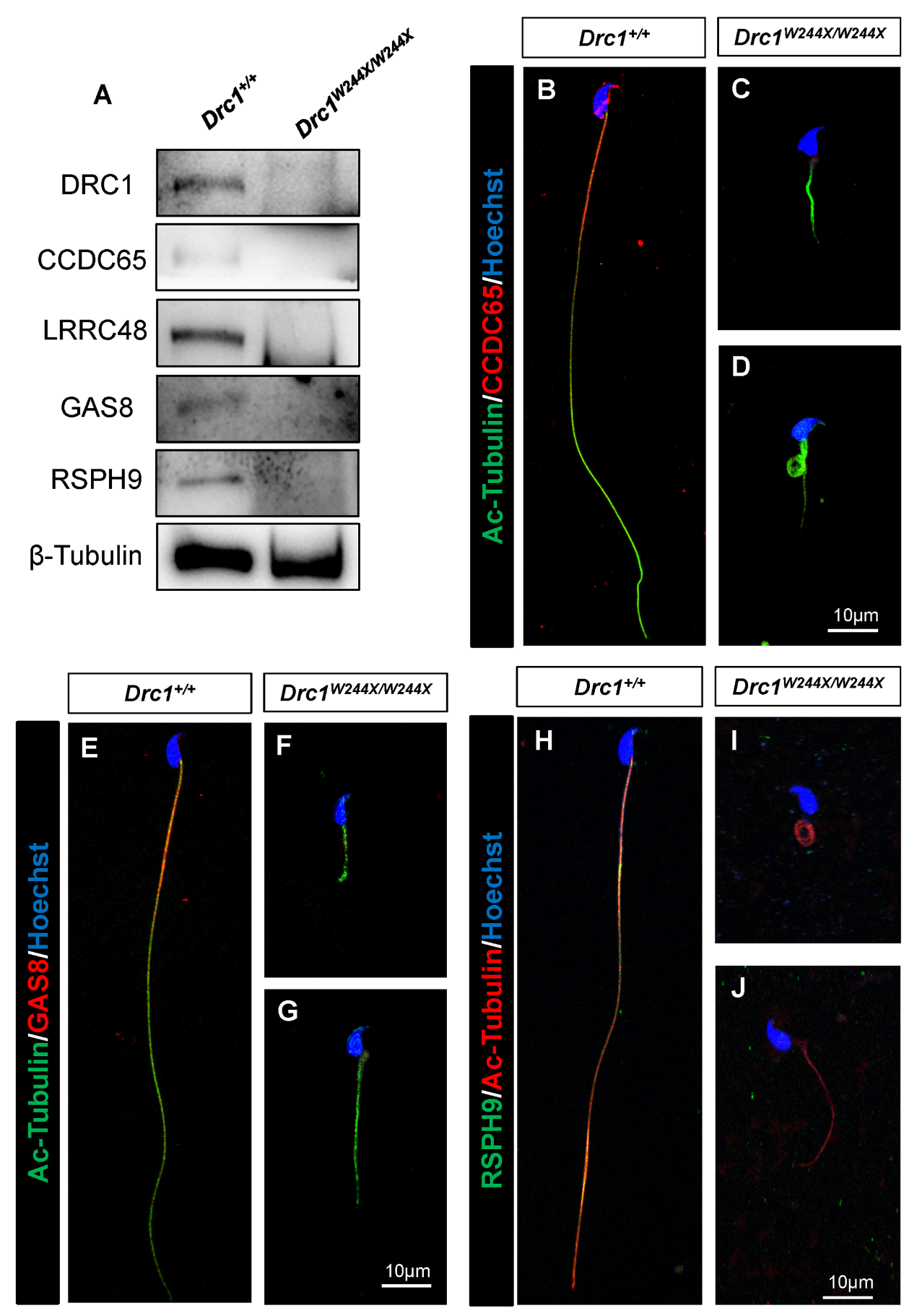


**Figure S10**

**DRC1 deletion affects the assembly of N-DRC in *Drc1*^W244X/W244X^ sperm.**

(A) Western blotting revealed that DRC1-4 and RSPH9 could not be detected in *Drc1*^W244X/W244X^ mature sperm. (B-D) An immunofluorescence analysis of acetylated-tubulin (green) and CCDC65(red) in wild-type and *Drc1*^W244X/W244X^ cells. (E-G) Immunofluorescence analysis of acetylated-tubulin (green) and GAS8 (red) in wild-type and *Drc1*^R554X/R554X^ cells. (H-J) Immunofluorescence analysis of acetylated-tubulin (red) and RSPH9 (green) in wild-type and *Drc1*^W244X/W244X^ cells.


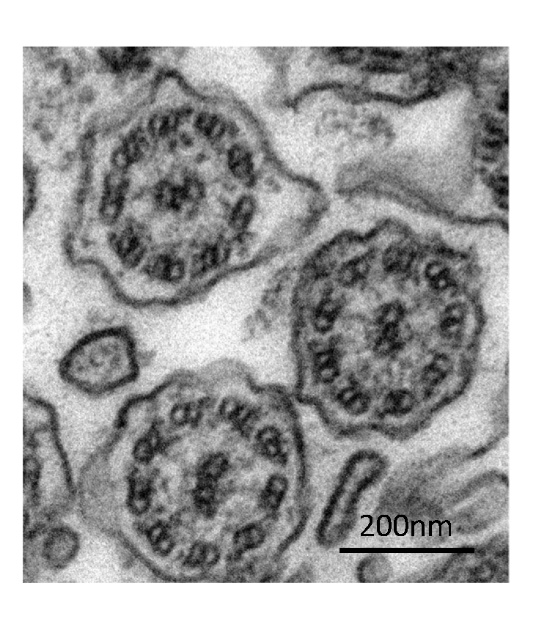


**Figure S11**

**TEM was used to assess respiratory ciliary cross-sections for *Drc1*^-/-^ in B6 background**


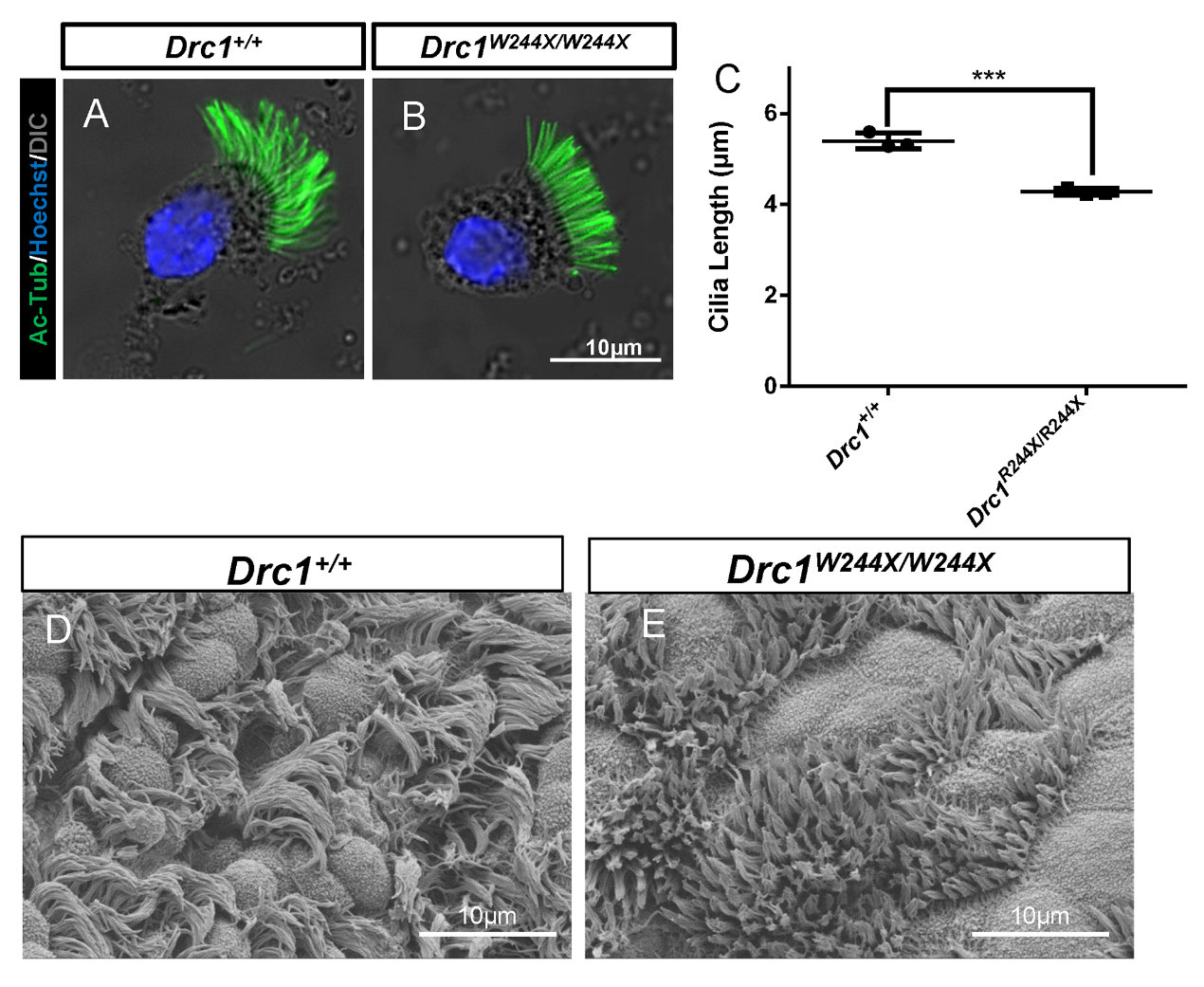


**Figure S12**

**The respiratory cilia of *Drc1*^W244X/W244X^ were significantly shortened**

(A-B) Acetylated-tubulin (green) was subjected to immunofluorescent staining in wild-type(A) and *Drc1*^W244X/W244X^ (B) samples. (C) The average length of isolated respiratory cilia from wild-type and *Drc1*^W244X/W244X^ subjects was analyzed, with each point corresponding to the average cilia length of one specimen (n=3). Data are represented as the mean ± SEM. (D-E) SEM was used to analyze respiratory cilia from wild-type(D) and *Drc1*^W244X/W244X^ (E) subjects.


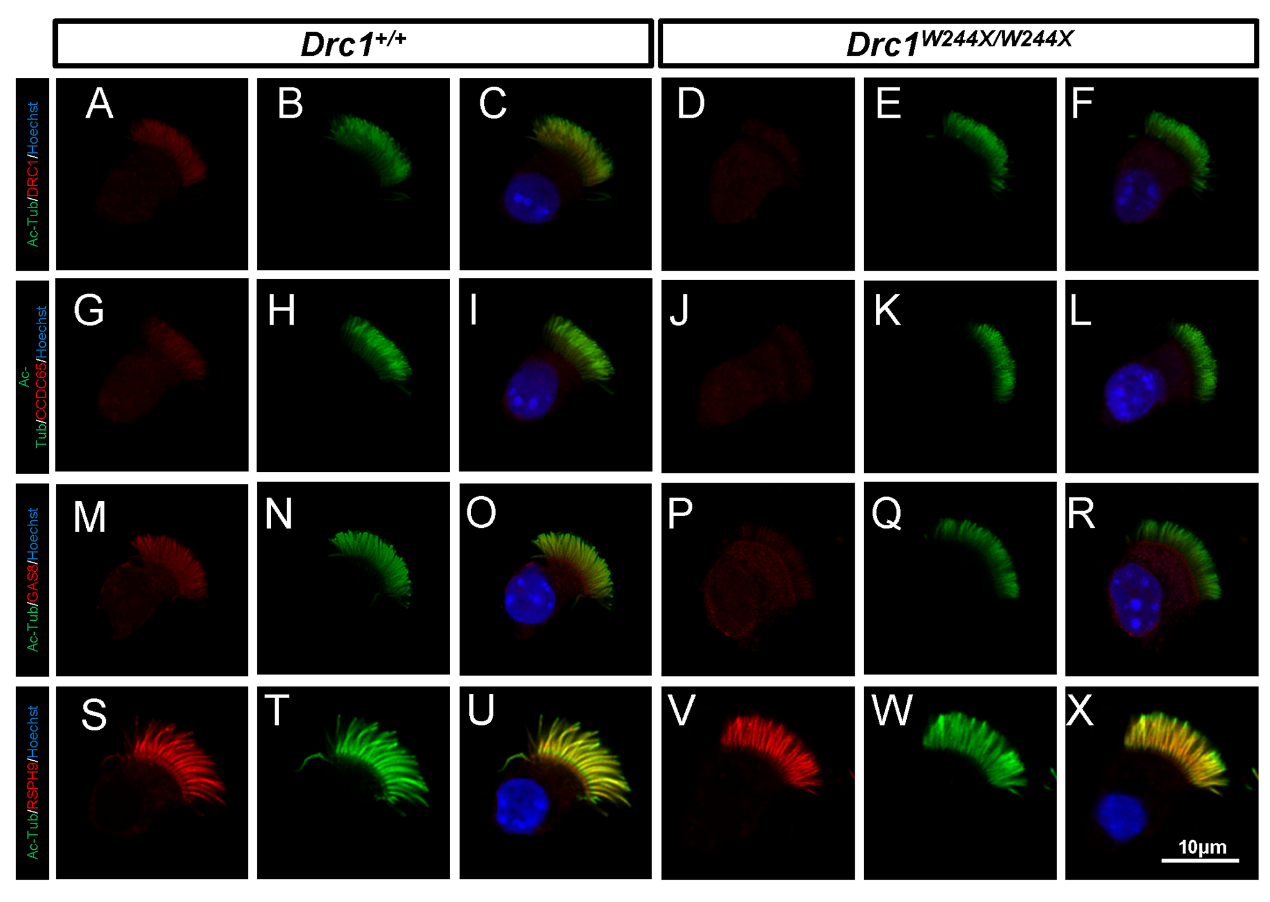


**Figure S13**

**Immunofluorescence analysis of respiratory cilia** **harboring the *Drc1*^W244X/W244X^ mutation**

(A-R) Wild-type and *Drc1*^W244X/W244X^ respiratory epithelial cells were dual-stained with an antibody marker for the ciliary axoneme (acetylated-tubulin, green) and an N-DRC antibody marker (DRC1, CCDC65, or GAS8, red). No N-DRC signal was detectable in *Drc1*^W244X/W244X^ cells. (S-X) Wild-type and *Drc1*^W244X/W244X^ respiratory epithelial cells were dual-stained with an antibody marker of the ciliary axoneme (acetylated-tubulin, green) and a radial spoke antibody marker (RSPH9, red). No difference in RSPH9 signal was observed between these cell types.


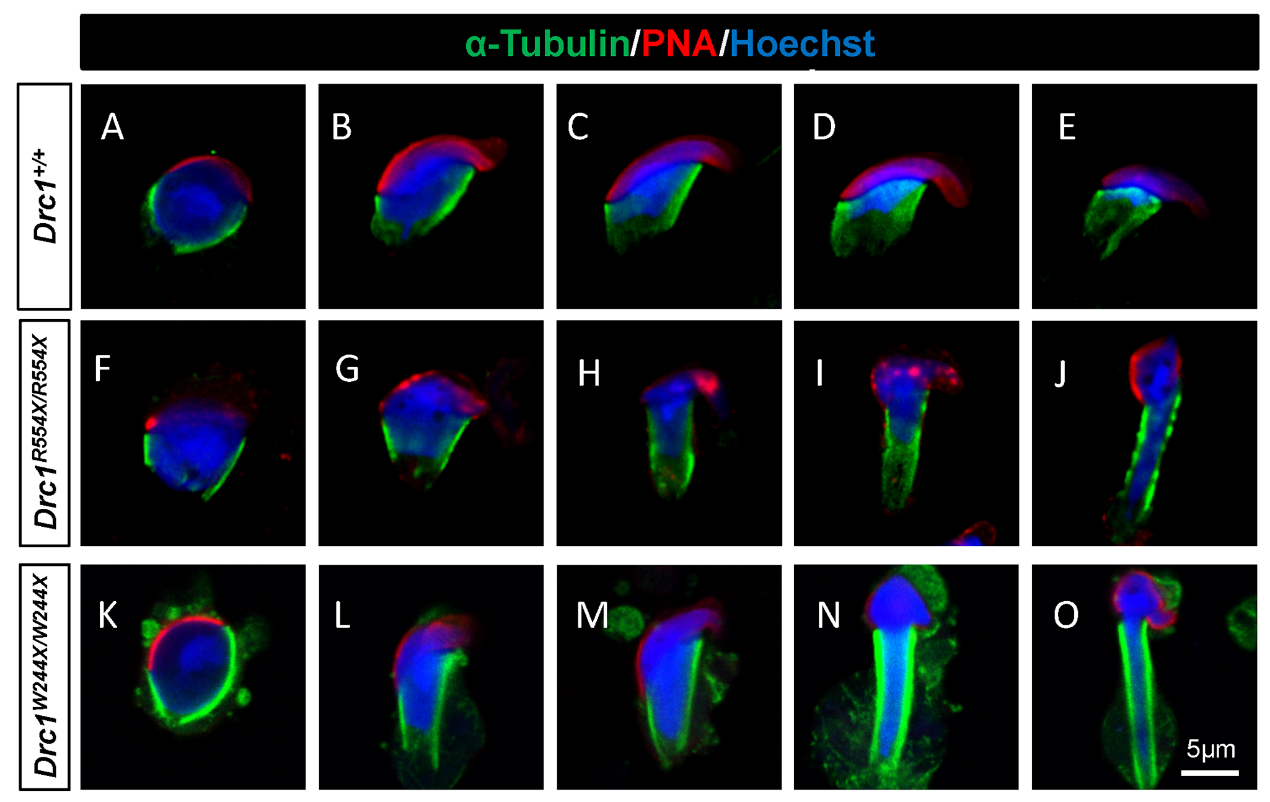


**Figure S14**

**An absence of DRC1 leads to nuclear deformation during spermiogenesis**

(A-O) Immunofluorescence staining for α-tubulin (green) and PNA (red) in wild-type (A-E), *Drc1*^W244X/W244X^*,* (F-J) and *Drc1*^R554X/R554X^ subjects (K-O). (A-E) α-Tubulin staining of manchette microtubules in elongating spermatids from WT mice. Elongating spermatids exhibit dynamic manchette changes, forming a sickle-shaped head. (F-J) A long and narrow manchette distribution around the nucleus was evident in elongating spermatids of *Drc1*^R554X/R554X^ and *Drc1*^W244X/W244X^ mice, leading to sperm heads of amorphous morphology.

**Table S1. Primers Used for Amplification and Verification of *DRC1* Mutations**

| Primer Names | Primer Sequences (5'-3') |
| --- | --- |
| DRC1 554-F | GTTCTGATGTGTGTGTCCCCAAG |
| DRC1 554-R | TGCTTTCTTCTCTCTGCACCTTGC |
| DRC1 80-F | GAAGTACAATGGAACCATTATTATTA |
| DRC1 80-R | CACAGAGCAGAAATGAACAA |

**Table S2. Primers Used for RT-qPCR**

| Primer Names | Primer Sequences (5'-3') |
| --- | --- |
| RT_DRC1-F | AGAAGAGAGCCGCCTGAAAC |
| RT_DRC1-R | CAGCTTCTCCAGTCTCTGCC |
| 18s-F | TAACGAACGAGACTCTGGCAT |
| 18s-R | CGGACATCTAAGGGCATCACAG |

Movie S1. Motility and movement of the sperm from the caput epididymis of wild-type male mice.

Movie S2. Motility and movement of the sperm from the caput epididymis of *Drc1*^W244X/W244X^ male mice.

Movie S3. Motility and movement of the sperm from the caput epididymis of *Drc1*^R554X/R554X^ male mice.

Movie S4. Ciliary motility of wild type tracheal epithelial cells.

Movie S5. Ciliary motility of *Drc1*^W244X/W244X^ tracheal epithelial cells.

Movie S6. Ciliary motility of *Drc1*^R554X/R554X^ tracheal epithelial cells.

Movie S7. Ciliary motility of *Drc1*^-/-^ in B6 background tracheal epithelial cells.
